# Asynchronous transcription and translation of neurotransmitter-related genes characterize the initial stages of neuronal maturation in *Drosophila*

**DOI:** 10.1371/journal.pbio.3002115

**Published:** 2023-05-19

**Authors:** Graça S. Marques, José Teles-Reis, Nikolaos Konstantinides, Patrícia H. Brito, Catarina C. F. Homem

**Affiliations:** 1 iNOVA4Health, NOVA Medical School, Faculdade de Ciências Médicas, NMS, FCM, Universidade NOVA de Lisboa; Lisboa, Portugal; 2 Department of Biology, New York University, New York, New York, United States of America; 3 Applied Molecular Biosciences Unit-UCIBIO, Departamento de Ciências da Vida, Faculdade de Ciências e Tecnologia, Universidade Nova de Lisboa, Caparica, Portugal; ICM, FRANCE

## Abstract

Neuron specification and maturation are essential for proper central nervous system development. However, the precise mechanisms that govern neuronal maturation, essential to shape and maintain neuronal circuitry, remain poorly understood. Here, we analyse early-born secondary neurons in the *Drosophila* larval brain, revealing that the early maturation of secondary neurons goes through 3 consecutive phases: (1) Immediately after birth, neurons express pan-neuronal markers but do not transcribe terminal differentiation genes; (2) Transcription of terminal differentiation genes, such as neurotransmitter-related genes VGlut, ChAT, or Gad1, starts shortly after neuron birth, but these transcripts are, however, not translated; (3) Translation of neurotransmitter-related genes only begins several hours later in mid-pupa stages in a coordinated manner with animal developmental stage, albeit in an ecdysone-independent manner. These results support a model where temporal regulation of transcription and translation of neurotransmitter-related genes is an important mechanism to coordinate neuron maturation with brain development.

## Abstract 2

Within hours of neuron birth, terminal differentiation genes (e.g. neurotransmitter-related genes) are transcribed, but not translated. This study in Drosophila reveals that these transcripts are only translated days later in a manner that is coordinated with animal stage, starting mid-pupation, just before synaptogenesis, but independent of ecdysone signaling.

## Introduction

Brain development is a complex process that requires the coordinated generation and maturation of thousands of neurons and glia cells. Ultimately, the formation of the right number and type of neurons and their synaptic connections results in the complex neuronal circuitry that will allow proper brain functioning.

During brain development, a small number of neural stem cells (NSCs) gives rise to the large neuronal diversity found in the adult brain (reviewed in, for instance, [1,2]). Studies in *Drosophila* have been fundamental to show how each neuronal fate is determined by the combination of several layers of transcription factors (TFs) and signaling pathways acting at the level of the NSC, intermediate progenitors, or even at the level of the differentiating neuron [3–9]. The core mechanisms of neural lineage progression and neuronal fate specification have been shown to be well conserved in mammals [8,10–13].

In *Drosophila*, neurons are formed in 2 waves. The first wave occurs in embryos to form the central nervous system of the embryo itself and of the larva (primary neurogenesis), while the second wave occurs in larva and early pupa and is responsible for the generation of the majority of the neurons that will populate the adult brain (secondary neurogenesis) [14]. Although secondary neurons are formed during a large developmental time window, neurons are described to remain immature until mid-pupal stages when synaptogenesis synchronously starts [15–19]. During this maturation period, neurons establish their morphological and electrophysiological properties and axon guidance occurs, ultimately allowing them to reach their target regions and connect with other neurons [20–23]. This process requires the coordinated expression of a combination of effector genes, responsible for the terminal differentiation of neurons, such as cell surface molecules, ion channels, and neurotransmitter (NT) receptors [18,24].

Several advances have been made in understanding how axon guidance occurs and how neurons choose and connect to their specific partners to form circuits [25]. However, to date, very little is known about the mechanisms that trigger neuronal maturation [26]. Interestingly, it was shown that the age-related transcriptomic diversity of *Drosophila* neurons is partially lost as early as 15 h after neuron birth, resulting in transcriptomic convergence in mature neurons [27]. This highlights the need to study young neurons as the transcriptomic profiles involved in the initial phases of neuron maturation can be quickly lost and may no longer be detectable in adult neurons. However, the transcriptomic datasets originated so far did not focus on young secondary neurons, not allowing for their clear distinction from mature neurons [18,27–35]. In this study, we characterized the transcriptional changes that lead to mature neurons in *Drosophila* larval central brain (CB) and ventral nerve cord (VNC) lineages. We devised a conditional genetic strategy to label, select, and sequence single-cell transcriptomes of secondary neuronal lineages, including only 0-h- to 12.5-h-old neurons (time relates to neuron birth, i.e., when the neuron is generated by a ganglion mother cell (GMC), it is considered to be 0 h old; 12.5 h later, this same neuron will be 12.5 h old). This time window was chosen as it is prior to neuron transcriptomic convergence [27]. The analysis of these young neurons allowed us, for the first time, to transcriptionally characterize the initial phases of neuron maturation.

We show that neuron maturation can be divided in 3 phases: a first phase, immediately after neuron birth, when neurons are specified but do not transcribe terminal differentiation genes; a second phase, which starts shortly after birth (<12 h), when neurons start transcribing, but not translating terminal differentiation genes such as the NT-related genes *vesicular glutamate transporter* (*VGlut*), *choline acetyltransferase* (*ChAT*), and *glutamic acid decarboxylase 1* (*Gad1*); and a third phase when these NT-related genes are translated in coordination with the animal developmental stage. We additionally show that translation inhibition or onset of NT-related gene translation occurs in an ecdysone-independent manner.

## Results

### Transcriptome sequencing of neural lineages including early-born neurons

**Fig 1:**
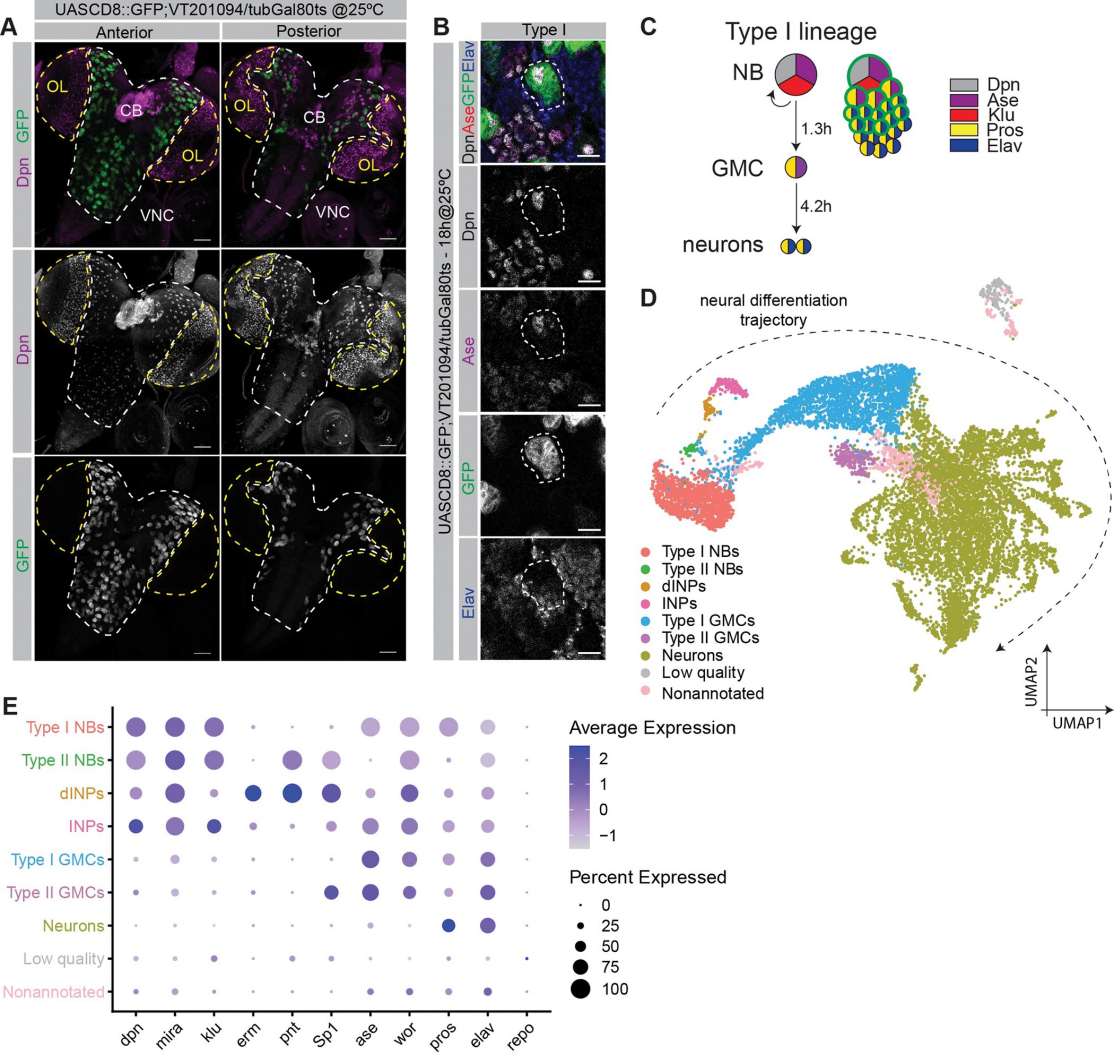
CB and VNC neural lineages identified by scRNA-Seq in wandering larvae brain. (**A**) VT201094-Gal4 driving expression of UAS::CD8-GFP. Expression occurs in neural lineages from CB and VNC, but not the OL. Views from anterior and posterior sides; Dpn (magenta), GFP (green); scale bar, 50 μm. (**B**) Close-up of one type I neural lineage, outlined, in the anterior side of CB; Dpn (white), Ase (magenta), GFP (green), Elav (blue); scale bar, 10 μm. (**C**) Schematic representation of type I neural lineages; cells colored by expression of markers as described; green outline indicates GFP expression and thus the cells in which VT201094-Gal4 drives expression in an 18-h time window; frequency of cell division is indicated in hours. (**D**) UMAP visualization of scRNA-Seq dataset composed of 12.7K cells of neural lineages from CB and VNC; clusters colored based on cell type annotation; dashed arrow indicates the direction of the neural differentiation trajectory. (**E**) Dot plot showing genes that were used to identify each cell type. Dot size indicates the percentage of cells expressing the gene in each cell type; color variation represents the average expression of the gene in each cell type. The data underlying this figure are contained within GEO database (accession number: GSE179763); see also S1 and S2 Figs. CB, central brain; dINP, developing INP; GMC, ganglion mother cell; INP, intermediate neural progenitor; NB, neuroblast; OL, optic lobe; scRNA-Seq, single-cell RNA sequencing; VNC, ventral nerve cord.

Most of the neurons that populate the adult *Drosophila* brain are generated during the second wave of neurogenesis. Secondary neurons are formed during larval and early pupal stages and then go through a maturation period to become functional. To characterize the transcriptional changes occurring in the early stages of neuron maturation, we performed scRNA-Seq (single-cell RNA sequencing) of third instar larval CB and VNC neural lineages. To ensure that only the lineages that originate secondary neurons were analyzed, we devised a conditional genetic strategy that was precisely controlled at a spatial and temporal level. We used the CB and VNC neuroblast (NB)-specific Vienna Tile Gal4 line#201094 (VT#201094) to drive the expression of CD8::GFP specifically in NBs (**Fig 1A**). As GFP protein is stable for several hours, it is inherited by the NB progeny, effectively labeling neural lineages. To control the time window of GFP expression, we included a temperature-sensitive (ts) tubGal80, which allows GFP expression at 25°C, while repressing it at 18°C [36,37]. With this conditional genetic system, we could precisely control when NBs start expressing GFP and thus label their progeny in the desired time frame.

We analysed staged wandering third instar larvae (equivalent to 105 h after larval hatching (ALH), at 25°C). These animals were raised at 18°C and shifted to 25°C 18 h prior to dissection to initiate GFP expression in NBs (**S1A Fig**). A type I NB divides every approximately 1.3 h to self-renew and to generate a GMC, which, after approximately 4.2 h, divides to form neurons and glia [38] (**Fig 1B and 1C**). Consequently, in an 18-h labeling window, the oldest neurons can be at most approximately 12.5 h old (18 h − 1.3 h − 4.2 h = 12.5 h), although most likely they are slightly younger as the expression of GFP protein under UAS-Gal4 takes some time to occur. Based on the durations of cell divisions, 18 h allow for type I NBs to divide up to 12 times, and for several GFP-positive neurons to be generated.

We focused our analysis on type I lineages as they constitute the majority of neural lineages in the CB and VNC (approximately 100 NBs per CB lobe, 25 NBs in each thoracic VNC hemisegment [3,39,40]). Additionally, an 18-h window allows for none or very few neurons to be labeled in type II lineages as those lineages have an extra intermediate progenitor state [38] (**S1B and S1C Fig**). We sorted the labeled cells by FACS, based on their size and GFP expression. Two samples were processed in parallel using the Chromium system (10x Genomics) and analyzed with standard Seurat pipeline [41,42]. Quality control (QC) metrics attested an overall good quality of the samples, such as low percentage of mitochondrial genes, and allowed us to set appropriate filters, resulting in a library of 12,671 single-cell transcriptomes and a total of 10,250 genes. The 2 samples were evenly scattered when visualized in low dimensional space using the uniform manifold approximation and projection (UMAP) algorithm [43], validating the good quality of our dataset (**S2A Fig**). We used Seurat’s graph-based clustering approach to select a resolution that was able to set apart one of the least frequent cell types in our dataset, the type II mature intermediate neural progenitors (INPs). This resolution was able to separate mature INPs from the remaining stages of nonmature INPs (grouped into a cluster named developing INPs (dINPs), as it includes the several progressive maturation stages of these progenitors; **S1C Fig**). This led to the identification of 39 clusters (**S2B Fig**). We used the expression pattern and average levels of known markers to identify the different neural lineage cell types in the UMAP (**Figs 1D, 1E and S2C**): All NBs express *dpn*, *mira*, *wor*, and *klu* (all gene symbols according to Flybase) [44–49]; however, type I NBs also express *ase*, while type II NBs do not (**Figs 1D, 1E and S2C**) [50]. Type II NBs and their lineage INPs and GMCs clustered separately and were identified by the expression of *Sp1* and *pnt* (**Figs 1D, 1E and S2C**) [51,52]. GMCs express *wor* and *ase*, but not *dpn* nor *mira* (**Figs 1D, 1E and S2C**) [44,46,50,53]. Neurons express *pros* and *elav* [54,55] but lack *dpn*, *mira*, and *wor* (**Figs 1D, 1E and S2C**).

The absence of cells expressing repo, a known glial marker [56,57], shows that this dataset does not include mature glia cells. Clusters characterized by the expression of ribosomal subunit genes were annotated as “low quality” [58], and clusters with no obvious expression of any neural markers were identified as “nonannotated.”

Overall, this dataset represents a snapshot of the different CB and VNC secondary larval neural lineages including only their young secondary neurons.

### Transcriptomic differences distinguish neurons by age

**Fig 2:**
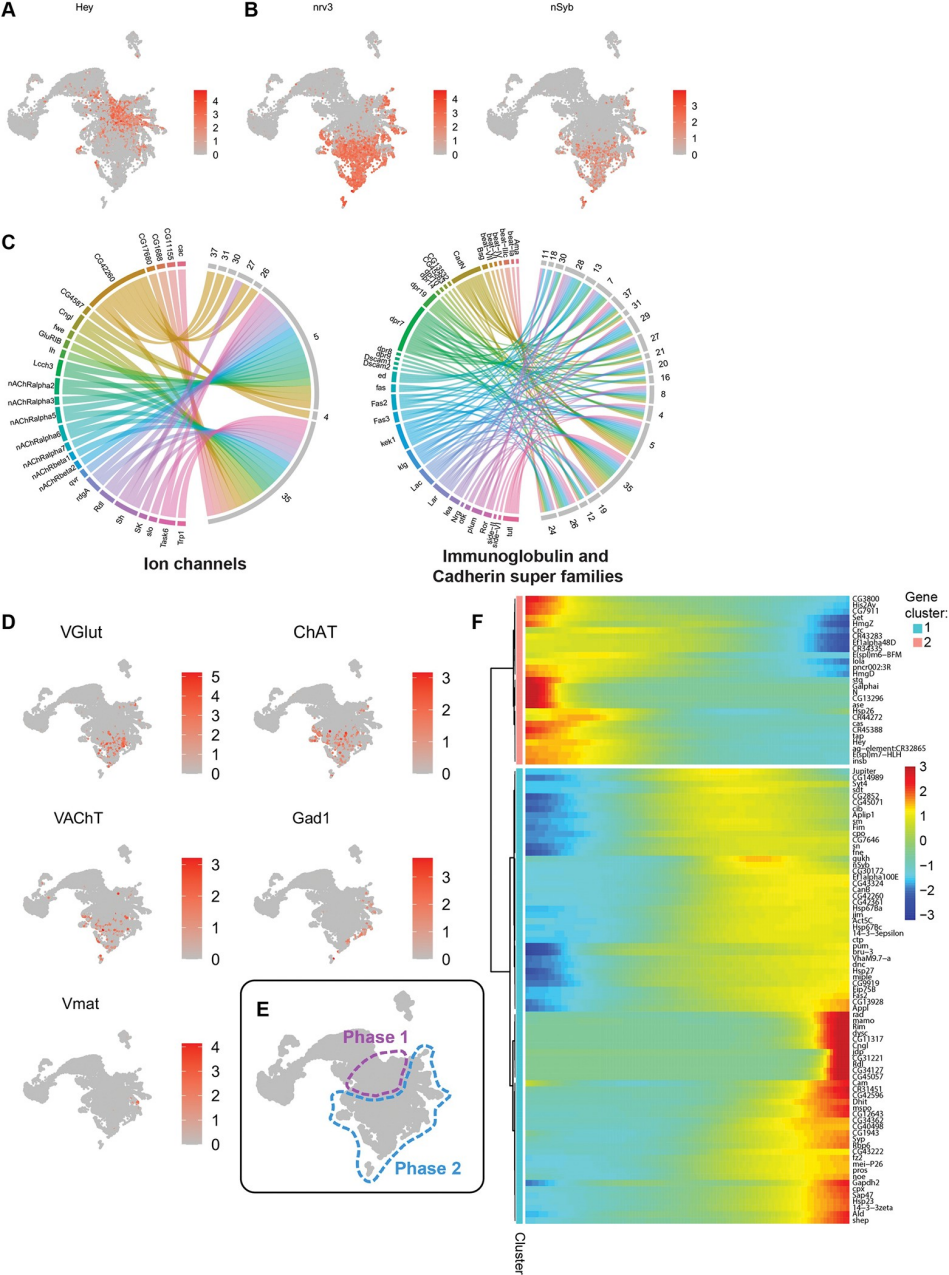
Transcriptomic differences in neurons identify 2 phases of maturation. (**A**) Feature plot for the marker of young neurons *Hey*. (**B**) Feature plots for the neuron maturation markers *nrv3* (right) and *nSyb* (left). (**C**) Chord diagrams showing the correspondence between ion channels (left) and immunoglobulin and cadherin super families (right) and the clusters in which these genes appear as top markers (top 5 markers). (**D**) Feature plots for neurotransmitter pathway identity markers (*VGlut*, *ChAT*, *VAChT*, *Gad1*, *Vmat*); these markers are predominantly expressed in older neurons. (**E**) Representative illustration of the proposed neuronal maturation phases in a UMAP plot: Phase 1 (younger/less mature) and Phase 2 (older/more mature). (**F**) Heatmap with the top 100 genes most differentially expressed throughout pseudotime in neurons; differential expression analysis performed with monocle. The data underlying this figure are contained within GEO database (accession number: GSE179763); see also S3 Fig and S1 and S2 Tables. *ChAT*, *choline acetyltransferase*; *Gad1*, *glutamic acid decarboxylase 1*; *nrv3*, *nervana 3*; *nSyb*, *neuronal Synaptobrevin*; UMAP, uniform manifold approximation and projection; *VAChT*, *Vesicular acetylcholine transporter*; *VGlut*, *vesicular glutamate transporter*; *Vmat*, *Vesicular monoamine transporter*.

Remarkably, the UMAP plot itself recapitulates the in vivo type I neural lineage progression order: NBs > GMCs > neurons (**Fig 1D**, dashed arrow). We have further validated this in our dataset by predicting the future state of each cell using the RNA velocity method (Velocyto; [59]). This method balances the abundance of unspliced and spliced mRNA variants to predict the future state of each cell. Moreover, it allows to calculate the local average velocity and represent it in a plot with an arrow. This Velocyto analysis predicted that cells progress from type I NBs, the less differentiated cells, to neurons, the more differentiated cells (**S3A Fig).** Moreover, Velocyto identifies age-related differences within the same cell type, as is the case for type I GMCs that seem to progress from young, closer to the NBs, to older, closer to neurons (**Figs 1D, S2B and S3A**). The same age progression was identified in the neuron population. Interestingly, even though all neurons in this dataset are younger than 12.5 h, both cluster analysis and the RNA velocity method suggest that within the neuronal population it is possible to identify different ages or degrees of maturation (**Figs 1D, S2B and S3A**). For instance, *Hey*, a known target of Notch that is transiently expressed only in Notch^ON^ early-born neurons [60] is expressed in approximately half of the neurons closest to the GMCs, i.e., the youngest neurons (**Fig 2A**). Conversely, *nrv3* and *nSyb*, known markers of differentiated neurons, are only expressed in neurons that are farther away from the GMCs in the UMAP plot (**Fig 2B**). We have additionally assayed for the expression of other markers of differentiated neurons as ion channels, immunoglobulins, and cadherin super families, which are essential for axonal development and neuronal circuit formation [61,62] (see Materials and methods for list details). Using these gene lists, we determined the correspondence between each cluster and the cluster top marker genes, which are genes present in these clusters and differentially expressed versus all others. Consistently, the neuronal clusters furthest from GMCs (clusters 5 and 35), predicted to be the older neurons in this dataset, have more ion channels and adhesion molecules as top marker genes **(Figs 2C and S3B**-outline). We also assessed the expression pattern of genes involved in the activity and biosynthesis of different NTs: *VGlut* (glutamatergic), *ChAT* and *VAChT* (cholinergic), *Gad1* (GABAergic), and *Vmat* (monoaminergic). This analysis revealed that these NT pathway genes are expressed in the neuron clusters furthest away from GMCs (**Fig 2D**), with the majority of neurons in our dataset being glutamatergic or cholinergic (**S3C Fig**). Notably, the young secondary neurons in our dataset mainly express a single NT pathway gene (**S3C Fig**), consistent with what has been described [29].

Thus, we propose that young neurons can be subdivided into 2 main groups depending on their degree of maturation. This distinction, correlated with neuronal age, is schematically represented in **Fig 2E**. We propose a “Phase 1” of maturation, which includes the very young immature neurons, which are specified and thus identified as neurons but are not yet transcribing terminal differentiation genes. Then, neurons progress into a “Phase 2” of maturation, when neurons start transcribing terminal differentiation genes.

To verify if there is indeed a transcriptional difference between younger/immature neurons and older/more mature ones, we did a subset of the neuronal population to analyse with Monocle. Monocle can identify differentially expressed genes throughout pseudotime and group them based on similar kinetic trends [63]. Within the neuronal population of our dataset, 2 major gene groups were identified based on their pseudotemporal expression pattern (**Fig 2F**). Gene cluster 2 represents a transcriptional profile composed by genes that are more expressed in neurons at the beginning of the pseudotime trajectory. A gene ontology (GO) analysis of these genes showed enrichment for terms associated with cell fate determination, regulation of neurogenesis, and neuron fate commitment (**S1 Table**). On the other hand, gene cluster 1 includes genes that are more expressed in neurons at the end of the pseudotime trajectory, meaning the oldest and more mature neurons in our dataset. Consistently, their GO term analysis revealed enrichment for terms related with synaptic transmission and NT regulation (**S2 Table**). This analysis suggests that young Phase 1 immature neurons are indeed transcriptionally different from Phase 2 neurons. Overall, the transcriptional analysis of young secondary neurons revealed that neurons begin their maturation process shortly after birth, a process marked by the unexpected expression of NT pathway genes and several other terminal differentiation genes as ion channels.

### Neurons in Phase 2 of maturation transcribe *VGlut* and *ChAT*, but no protein can be detected

**Fig 3:**
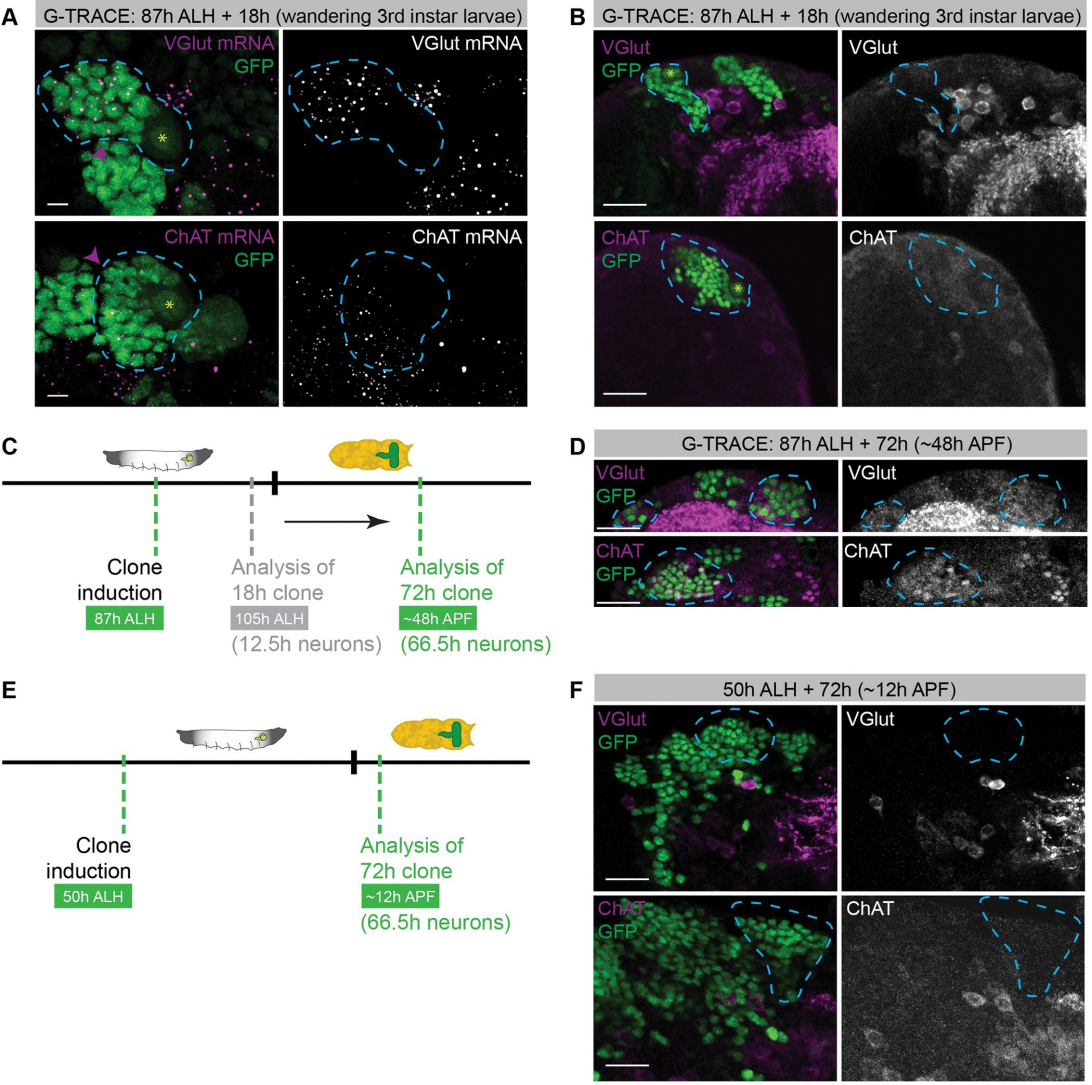
The timing of VGlut and ChAT protein detection depends on animal age. (**A, B**) 18-h clones induced at 87 h ALH with the VT201094-Gal4 driver using G-TRACE; VT201094-Gal4;tubGal80ts was used to permanently label with GFP NB-derived lineages using G-TRACE; clones were analysed at 105 h ALH (wandering third instar larvae); oldest neurons in the clone are approximately 12.5 h old. (**A**) smFISH against VGlut or ChAT. Individual mRNA molecules are displayed as magenta dots; neural cells are labeled with nuclear GFP (green). Dashed line delimitates a neural clone; pink arrowhead indicates the closest cell to the neuroblast where mRNA is visible; yellow asterisk identifies neuroblast. Z-projection from 21 slices with 0.25 μm interval, resulting in a total z range of approximately 5 μm (roughly the size of a neuron); scale bar, 5 μm. (**B**) Immunofluorescence images for VGlut or ChAT antibody staining (magenta). Neural cells in clones are labeled with nuclear GFP (green); outlines indicate examples of lineages with GFP-positive cells; yellow asterisk identifies neuroblast. Scale bar, 20 μm. (**C, D**) 72-h clones induced at 87 h ALH and analysed at 159 h ALH (approximately 48 h APF) using G-TRACE; oldest neurons in the clone are approximately 66.5h. (**C**) Schematic representation of the temporal strategy used to induce 72-h clones in neural lineages represented in green. Timings used for the scRNA-Seq dataset (18-h clones induced at 87 h ALH and analysed at 105 h ALH) are represented in grey. (**D**) Immunofluorescence images for Vglut or ChAT antibody staining (magenta). VT201094-Gal4 drives nuclear GFP (green) expression in NBs, daughter cells generated by NBs during clone period inherit GFP expression. Outlines indicate clones; scale bar, 20 μm. (**E, F**) 72-h clones induced at 50 h ALH and analysed at 122 h ALH (approximately 12 h APF) using G-TRACE; oldest neurons in the clone are approximately 66.5 h. (**E**) Schematic representation of temporal strategy used to induce clones in neural lineages. (**F**) Immunofluorescence images for VGlut or ChAT antibody staining (magenta). VT201094-Gal4 was used to drive nuclear GFP (green) expression in NBs; outlines indicate neurons clones; scale bar, 20 μm. See also S4 Fig. ALH, after larval hatching; APF, after puparium formation; ChAT, choline acetyltransferase; NB, neuroblast; scRNA-Seq, single-cell RNA sequencing; smFISH, single-molecule fluorescence in situ hybridization; VGlut, vesicular glutamate transporter.

The expression of NT pathway genes, ion channels, and other terminal differentiation genes in secondary neurons in larval stages was surprising as these neurons will only initiate synaptogenesis days later in pupal stages [17]. Hence, to further validate our observations done by scRNA-Seq, we assessed the presence of mRNA molecules in vivo by single-molecule fluorescence in situ hybridization (smFISH). We analysed the mRNA expression pattern for the most abundant NT pathway markers VGlut, ChAT, and Gad1. We labeled cells using a similar strategy as to previously described, raising the animals at 18°C and shifting them to 25°C 18 h before dissection, at an equivalent developmental time of 105 h ALH (**S4A Fig**). In this set of experiments, we used a permanent GFP labeling strategy to allow us to label the secondary neurons formed and to track these neurons until later time points. A clonal strategy is required to identify secondary neurons in the larval and pupal CB, as in the CB secondary neurons coexist with primary neurons, which were made during embryogenesis and are already mature at these stages. This strategy to label secondary neurons uses a ubiquitin promotor to control Stinger expression (a nuclear-localized EGFP) upon removal of an FRT cassette by a *UAS*-FLP [64]. The described smFISH analysis detected *VGlut*, *ChAT*, and *Gad1* mRNA molecules in the progeny generated in this 18-h period, showing that indeed early-born secondary neurons (<12.5 h old) can already transcribe these terminal differentiation genes (**Figs 3A, S4A, and S4B**). Based on our transcriptomic analysis, these 18-h clones should also contain Phase 1 neurons that have not yet started NT gene transcription. To quantitatively determine the amount of mRNA in young versus older neurons, and as antibodies for lineage fate markers do not function well with the smFISH protocol used, we devised a quantification strategy based on the distance from the cell to the mother NB. NB lineages are stereotypical and as cells do not migrate, cells closer to the mother NB are younger than cells further away, which are pushed inward the brain with the birth of new cells. Consistently, in the cells closest to the NB (Region 1/green; **S4C Fig**), the number of smFISH spots representing the abundance of mRNA of *ChAT* or *Gad1* was significantly lower than the number of spots present in the cells further away from NBs (Region 2/magenta; **S4C and S4D Fig, S1 Data**). The quantification of the number of smFISH spots of *VGlut* in younger versus older progeny was difficulted as independent lineages were hard to separate and thus only a small number of cells could be accurately counted. Nonetheless, this quantification shows that secondary neurons express *VGlut* mRNA. Additionally, the cells further away from the NB, predicted to be older/Phase 2 neurons, tend to have higher number of spots when compared to cells closer to the NB, although this difference was not statistically significant (**S4D Fig, S1 Data**).

Next, we wanted to determine if the presence of mRNA in the Phase 2 neurons is already accompanied by the expression of the respective protein. Therefore, we again generated 18-h clones but this time tested for the presence of VGlut or ChAT proteins with available antibodies. Interestingly, none of the secondary neurons (GFP^+^) generated in the 18-h window is stained with either VGlut or ChAT antibody, indicating that these proteins are still not expressed (**Fig 3B**, outlines). These results indicate that even though the oldest neurons in our dataset (Phase 2) already express *VGlut* and *ChAT* mRNA, these molecules are either not yet being translated into protein or that the levels of the proteins made are somehow kept bellow detection limit in these maturing neurons.

### Initiation of VGlut and ChAT protein detection is coordinated with animal developmental stage rather than neuron age

The fact that *VGlut* and *ChAT* transcripts are present, but their proteins are absent, suggests that there is a mechanism delaying the expression or accumulation of these proteins in maturing neurons. To understand what determines the time point when VGlut/ChAT protein is first detected in maturing neurons, we tested whether (1) the presence of VGlut/ChAT protein is dependent of neuron age and requires neurons to be older/of a certain age; or (2) VGlut/ChAT protein levels are coordinated with animal development starting to be translated or accumulated only at a certain stage of brain development. In order to test these hypotheses, we again used a permanent labelling strategy to fluorescently label neural lineages, thus labelling neurons from their birth, and follow them until a certain age. We have previously shown that in third instar larvae, neurons younger than 12.5 h VGlut/ChAT protein cannot be detected (**Fig 3B**). To test if in older neurons there is already VGlut/ChAT protein, we have allowed these neurons to age for a longer time. To do this, we induced clone formation at the same time as previously (87 h ALH), but instead, we let them develop for 72 h. Hence, the older neurons would be up to 66.5 h old, and the animal would be 159 h ALH (approximately 48 h after puparium formation; APF; **Fig 3C**). In these clones, coexpression of GFP and either VGlut or ChAT protein is detected in several neurons cell bodies (**Fig 3D,** outline).

However, these results still fit both hypotheses, since neurons may have VGlut/ChAT protein because they are older than in the previous clones, or simply because the animal itself is older and closer to the onset of synaptogenesis [17]. In order to uncouple neuronal age from the animal’s age, and tease apart these 2 options, we generated a clone for the same duration of 72 h but starting at an earlier time in animal development. We have thus induced clone formation at 50 h ALH (**Fig 3E**). This new timeline still allows neurons to age up to 66.5 h old, but the animal itself, although still a pupa, would be only approximately 12 h APF (122 h ALH). Interestingly, in these clones, there is no codetection of GFP and VGlut or ChAT (**Fig 3F**). Hence, the presence of VGlut and ChAT protein is not necessarily triggered by neuronal age itself, being rather dependent on the age of the animal.

Overall, our results indicate that the detection of protein for NT pathway genes in maturing neurons depends on the animal developmental stage, representing what we propose to be a third phase of neuronal maturation.

### *VGlut*, *ChAT*, and *Gad1* are transcribed days before protein is present also in optic lobe neurons

To determine if the observed pattern of expression of NT pathway genes is also conserved in other brain regions and neuron types, we analysed maturing neurons in the optic lobe. The neurons that compose the adult visual system are all generated in post-embryonic stages, i.e., they are all secondary neurons [65], and establish synapses in mid-pupal stages at approximately 55 h APF [15,19,66].

**Fig 4:**
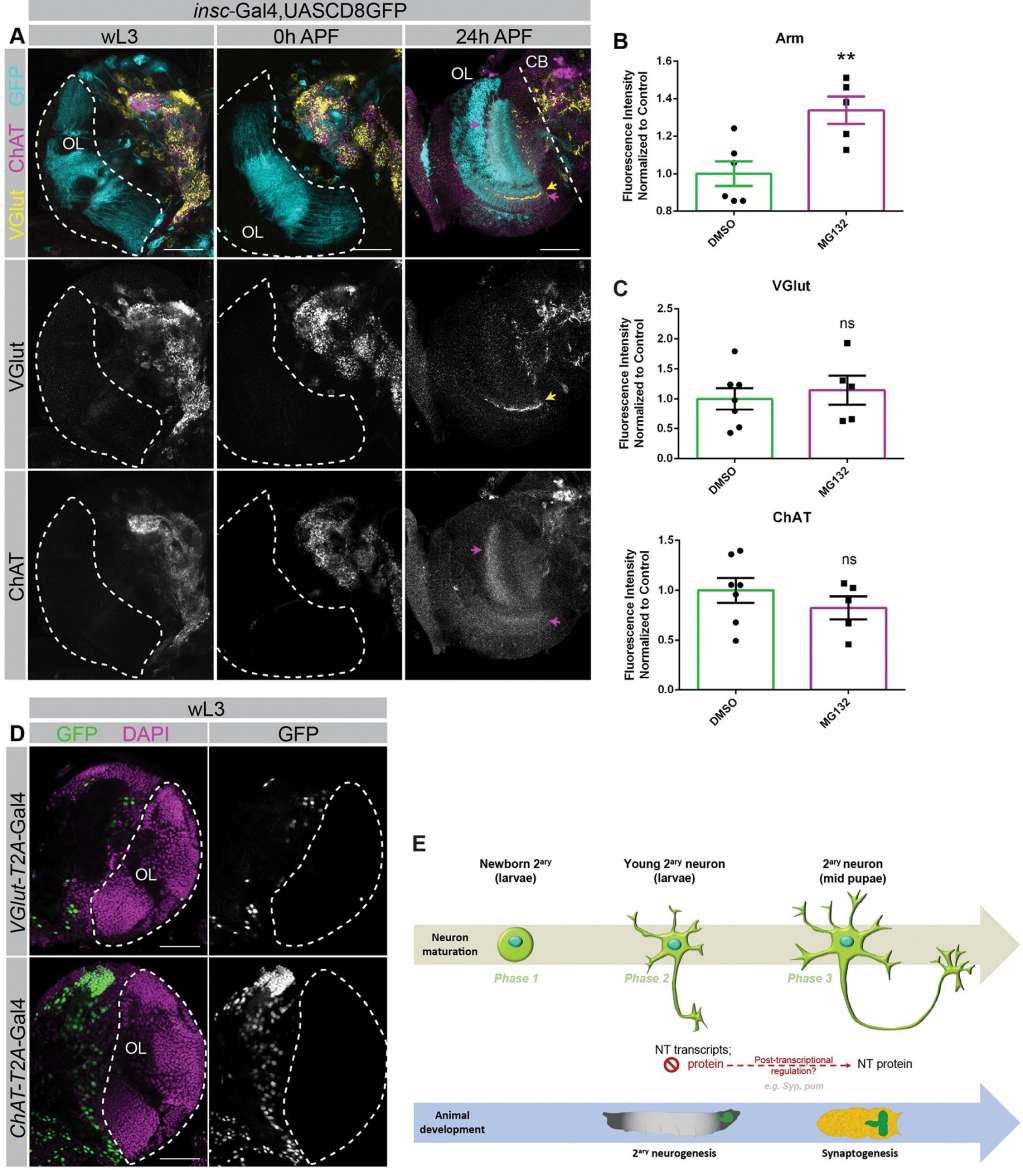
VGlut and ChAT proteins are not detected in wL3 OL. (**A**) *insc*-Gal4,UAS-CD8::GFP was used to label NB lineages in the OL. GFP(cyan), VGlut (yellow), and ChAT (magenta) protein expression in the OL at wL3 stages, 0 h APF, and 24 h APF. Insc-Gal4 is expressed in both OL and CB regions; OL is delimitated by a dashed line. Arrows in 24 h APF panel refer to VGlut protein (yellow) or ChAT protein (magenta) expression. Scale bar, 50 μm. (**B, C**) Mean fluorescence intensity measured in wL3 raised in food supplemented with MG132 diluted in DMSO, or in DMSO alone (control). (**B**) Arm mean fluorescence intensity was measured in OL neuroepithelia (control, *n =* 6; MG132, *n* = 5). (**C**) VGlut and ChAT mean fluorescence intensity was measured in medulla neurons (control, *n* = 7; MG132, *n* = 5). Plotted data were normalized to the mean of the control. Data shown as mean ± SEM; statistical analysis was done using unpaired two-tailed *t* test; **P value < 0.01, ns = nonsignificant. The data underlying this figure are contained within S3 Data. (**D**) UAS-*Stinger*::GFP (green) expression driven by *VGlut*-T2A-Gal4 or *ChAT*-T2A-Gal4 in wL3 stages. DAPI (magenta). OL delimited by dashed line. CB located to the left of the OL. Scale bar, 50 μm. (**F**) Model for the asynchronous onset of *VGlut* and *ChAT* transcription and translation during the first phases of neuron maturation. See also S4 Fig. APF, after puparium formation; Arm, Armadillo; CB, central brain; ChAT, choline acetyltransferase; NB, neuroblast; OL, optic lobe; VGlut, vesicular glutamate transporter; wL3, wandering L3.

We have thus stained larval and pupal optic lobe regions with antibodies against VGlut or ChAT. Since antibodies anti-Gad1 are not available, we have assayed Gad1 expression utilizing an allele of Gad1 endogenously tagged with EGFP [67,68]. This analysis revealed that during third instar larval stages, no VGlut, ChAT, or Gad1 are detected in the entire region of the optic lobe (**Figs 4A**, wL3 and 0 h APF; **S4E**, wL3). VGlut, ChAT, and Gad1 proteins were only detected, albeit at still low levels, at approximately 24 h APF (**Figs 4A**, 24 h APF; **S4E Fig**, 24 h APF). As a large fraction of optic lobe neurons are interneurons, whose cell bodies and projections remain within the optic lobe [65], the absence of NT proteins in the entire optic lobe region additionally shows that there is no protein in neither neuron cell bodies or in their extensions. As Gad1 is also normally expressed in cell bodies [69–71], this experiment also supports that NT protein absence in young stages is not related to the status of vesicle formation or maturation. To determine if NT pathway genes are also transcribed in the optic lobe during the stages when no protein is detected, we have reanalysed the single-cell transcriptome dataset generated in [18] where the single-cell transcriptome of the optic lobe was sequenced from 0 to 96 h APF in 12-h intervals. This analysis showed that optic lobe neurons transcribe NT pathway genes at 0 h APF (normalized average expression ranging from 0.21 to 5.74; **S2 Data**), several hours before protein starts being detected in mid-pupal stages.

One possibility is that protein only accumulates to detectable levels when the levels of transcripts reach a certain threshold, and this is why protein is not visible up to 24 h APF. To test this hypothesis, we selected a subset of neurons that represent glutamatergic, cholinergic, or GABAergic neurons and for which counts for the corresponding NT transcript can be detected in all time points. We have thus selected 2 glutamatergic neurons (VGlut^+^; L1, Mi9), 2 cholinergic neurons (ChAT^+^; L5, Mi1), and 2 GABAergic neurons (Gad1^+^; Dm10, Mi4) (**S4F Fig, S2 Data**). This analysis revealed that NT genes are expressed at constant levels from early pupal stages (0 h APF) to approximately 48 h APF (**S4F Fig, S2 Data**). Although the expression levels of VGlut were found to be more variable, the constant levels of Gad1, ChAT (**S4F Fig, S2 Data**) show that there is not a consistent increase in the levels of transcripts preceding the start of protein detection in optic lobe neurons (approximately 24 h APF; **Fig 4A**). So, an increase in transcription levels cannot by itself justify the detection of protein at 24 h APF. The observed increase in NT gene transcription levels from approximately 48 h APF onward has been previously reported, and it is thought to be related to neuronal activity [18].

Overall, these results show that NT pathway genes are also transcribed in larval optic lobe neurons days before protein is detected, showing that this is a conserved mechanism occurring in maturing neurons in multiple brain regions. These results further show that the beginning of VGlut, ChAT, or Gad1 protein detection does not occur as a consequence of transcript accumulation.

### Absence of VGlut and ChAT proteins in young maturing neurons in larval stages cannot be explained by low levels of translation or proteasomal protein degradation

To try to understand the mechanism that temporally regulates the levels of VGlut or ChAT protein in the different phases of maturing neurons/animal stages, we next tested several hypotheses:

One interesting hypothesis is that there is translation of VGlut and ChAT during larval and young pupal stages but that the protein formed during these early stages is quickly degraded. Later in mid-pupal stages, these proteins would no longer be degraded and would therefore accumulate. This would be interesting on its own as it would indicate that there is an active mechanism to degrade these proteins and ensure that NT protein levels are maintained at low levels until mid-pupal stages potentially coordinated with synaptogenesis. To test this hypothesis, we blocked protein degradation by inhibiting the proteasome with the well-described proteasome inhibitor MG132 [72]. We treated larvae with MG132 as previously described [73] to determine if proteasome inhibition would lead to accumulation of VGlut or ChAT protein in larval optic lobes (when and where normally no protein is detected). To ensure that the treatment with MG132 was effective, we quantified the levels of Armadillo (Arm) in optic lobe neuroepithelia as a positive control. *Drosophila* Arm is targeted for destruction by the proteasome unless stabilized by extracellular Wnt signals [74]. Confirming that inhibition of the proteasome by MG132 was effective, Arm was significantly accumulated in the neuroepithelia (**Fig 4B, S3 Data**). However, proteasome inhibition with MG132 did not lead to the accumulation of VGlut or ChAT in optic lobe (**Fig 4C, S3 Data**; analysis done in medulla neuron region that contains both glutamatergic and cholinergic neurons) [65]. In addition, we have inhibited the proteasome by knocking down several subunits of the proteasome using previously validated RNAi lines [75]. Consistent with the results obtained with MG132, knockdown of these proteasome subunits in optic lobe neurons did not cause accumulation of VGlut of ChAT (**S4G Fig, S4 Data**). These results indicate that NT proteasomal protein degradation is not the mechanism responsible for the undetectable levels of protein in young neurons in larval brains.

To further test if there are low levels of translation of NT proteins in larval stages that might be below the detection limit of antibodies, we have used a transgenic line in which a T2A-Gal4 is knocked-in downstream of endogenous VGlut or ChAT (VGlut-T2A-Gal4 and ChAT-T2A-Gal4; [76]). 2A peptide-linked polycistronic vectors can be used to express multiple proteins from a single open reading frame; a cleavage event at the 2A sequence then separates the individual proteins. Thus, if there is translation of VGlut or ChAT proteins, then Gal4 would also be translated. Gal4, a TF originally cloned from yeast, contains a DNA-binding domain (DBD) and a transcription activation domain (AD), and binds to a specific sequence, UAS (upstream activation sequence) [77]. The Gal4/UAS system results in the expression of downstream genes at much higher levels than endogenous tissue-specific promoters [78]. Therefore, this amplification process generates high levels of gene expression. We have thus crossed these NT-T2A-Gal4 to UAS-Stinger-GFP (nuclear fast-maturing, insulated, stable enhanced-GFP; [79]) to determine if there are low levels of translation occurring. The brightness and stability of this Stinger-GFP protein potentiates for even a low number of molecules to be detected.

We have performed these analyses in the optic lobe in larval stages, when normally no NT protein can be detected with antibodies. However, we have observed no GFP expression in VGlut-T2A-Gal4; UAS-Stinger-GFP or ChAT-T2A-Gal4; UAS-Stinger-GFP animals (**Fig 4D**, outline). As a confirmation that these lines are functioning, we can detect GFP signal in the mature primary neurons in the larval central brain (**Fig 4D,** to the left of the dashed lines). Combined, all these results provide strong evidence supporting that VGlut and ChAT are not translated in secondary neurons in larval stages.

Interestingly, this suggests that there is a delay in translation initiation of these NT genes and that translation then starts in mid-pupa stages in a coordinated manner with animal development.

### EcR does not have a role in regulating the timing of VGlut translation

The coordinated translation of NT pathway genes in mid-pupa stages could, for instance, be a mechanism to synchronize the maturation of neurons that are synaptic partners. The steroid hormone ecdysone is a coordinator and central regulator of developmental transitions in *Drosophila*, and it has been shown to be involved in the regulation of neural progenitor fate [80,81]. We have thus hypothesized that ecdysone could be a regulator of NT gene translation timings during neuron maturation. There are several pulses of ecdysone during both larval and pupal development [82,83], making it a potential regulator of NT gene translation inhibition in larva or translation initiation in pupa.

To study the possible role of ecdysone in regulating translation initiation of NT pathway genes, we inhibited ecdysone signalling by expressing the well-established dominant negative form of the ecdysone receptor (EcR; EcRDN) in neural lineages. For these analyses, we focused on CB young secondary glutamatergic neurons, which represent the biggest NT family in our dataset (**S3C Fig**).

We first tested if ecdysone is necessary for the inhibition of VGlut translation in CB secondary neurons during larval stages. We have thus again generated 18-h GFP^+^ clones in third instar larvae, now coexpressing EcRDN. However, the inhibition of ecdysone signalling in these lineages did not cause premature expression of VGlut protein expression at 105 h ALH (**S5A and S5A’ Fig**). These results show that ecdysone is not necessary for the inhibition of VGlut translation in young secondary larval neurons. Next, we tested if ecdysone was regulating NT gene translation initiation in pupal stages. To analyse this, we expressed EcRDN in neurons using the VT201094 driver for 72 h, assessing VGlut protein expression at approximately 48 h APF, a time point when normally VGlut protein expression is already started. The inhibition of ecdysone signalling in these lineages did not prevent initiation of translation of VGlut (**S5B and S5B’ Fig**).

As the VT201094 driver is expressed in NBs, and to ensure that EcRDN expression was being maintained at high levels in neurons, we have additionally expressed EcRDN with *elav*-Gal4. As *elav*-Gal4 is expressed in all neurons, we have assayed the total volume of VGlut-positive neurons in both control and EcRDN larval or pupal brains and observed no significant differences (**S5C, S5D, and S5E Fig, S5 Data**). Morphologically, we have also observed no difference in the pattern of VGlut expressing neurons (**S5D and S5D’ Fig**).

As an alternative way to knock down ecdysone signaling, we have also ablated the production of ecdysone by inducing apoptosis of the prothoracic gland (PG). The PG is responsible for the synthesis of ecdysone. To eliminate the PG, we used a genetic combination to induce the expression of the proapoptotic gene, *grim* [84], specifically in the PG (*Phantom*-Gal4, a PG-specific Gal4) in a temperature-sensitive manner (UAS-Grim × phmGal4::tubGal80*ts*) [85]. Animals are normal when reared at 18°C, but when the temperature is shifted to 25°C, the expression of *grim* in the PG promotes its apoptosis [85,86], blocking the production of ecdysone. We have induced the ablation of the PG gland in white pupa (0 h APF), which corresponds to a stage prior to presence of NT proteins, to determine how this affects NT protein in mid-pupal stages. Consistent with the results obtained with EcRDN, at 48 h APF, VGlut protein is normally expressed in brains without PG (PGX; **S5F and S5G Fig, S5 Data**).

Overall, these results indicate that ecdysone signalling is not necessary for the inhibition of NT translation in larval stages nor is it necessary to trigger NT protein translation in pupal stages.

## Discussion

This study has allowed us to characterize in detail the first stages of neuron maturation, revealing that within a few hours from birth (<12.5 h old), neurons have transcriptional profiles that reflect their different ages and consequently different degrees of maturation. Our analysis of CB and VNC young secondary neurons led us to propose a model that includes a “Phase 1” of maturation where newly born neurons do not transcribe NT pathway genes and other terminal differentiation genes. This is quickly followed by a “Phase 2” of maturation when neurons start transcribing NT pathway genes, ion channels, and other terminal differentiation genes. We further show that in these young neurons, *VGlut*, *ChAT*, and *Gad1* mRNA are, however, kept untranslated. Afterwards, approximately at 48 h APF, a “Phase 3” of maturation starts, when the neuron begins translation of VGlut, ChAT, and Gad1 in a manner that is coordinated with animal and brain development rather than with the age of the neuron itself (**Fig 4E**). The dynamics of *VGlut*, *ChAT*, and *Gad1* mRNA and protein are also similar in maturing optic lobe neurons, showing that this is a conserved mechanism in multiple brain regions. As the optic lobe is only composed by secondary neurons, its analysis permitted the examination of the complete neuron to show that there is no VGlut, ChAT, or Gad1 protein neither in neuron cell bodies nor in their extensions. As the start of protein detection could not be explained by an increase in NT pathway gene levels, we tested several hypotheses to try to explain the asynchronous detection of transcripts and protein in maturing neurons. One hypothesis was that these proteins were translated in these early developmental stages but quickly degraded. Interestingly, proteasomal NT protein degradation does not seem to be the mechanism responsible for the undetectable levels of NT proteins in larval neurons. Another possibility was that VGlut and ChAT were translated at low levels, below immunofluorescence detection limit. To test this, we used a translation reporter (NT-T2A-Gal4). If there were translation of these NTs, Gal4 would also be translated and would lead to the expression of a nuclear-eGFP. As the Gal4/UAS system results in the amplification of expression signal, and we have used a bright and stable GFP, the translation of a few molecules of NT genes would more likely be observed. However, there was no GFP signal in larval optic lobes, indicating that there is no translation of VGlut or ChAT in these Phase 2 maturing neurons. This experiment also corroborates that these NT proteins are not being degraded by other nonproteasomal methods, as even if they were degraded, Gal4, which is a yeast protein and known to be stable in *Drosophila*, would still be able to activate the expression of UAS-Stinger-GFP. Combined, these results suggest that there is a delay in translation initiation of these NT genes in Phase 2 neurons and that their translation then starts in mid-pupa stages in a coordinated manner with animal development (Phase 3). We can, however, not exclude that very few molecules are translated and not detected, but even so, these results interestingly show that there is an active mechanism to keep NT genes not translated or translated at very low levels in maturing neurons until the animal reaches a predetermined developmental stage.

At this point, we can only suggest this model for some neuroactive molecules, more specifically for VGlut, ChAT, and Gad1, which by itself is already relevant as glutamatergic and cholinergic neurons alone make up to over 60% of the neurons in the adult *Drosophila* brain [32]. In the future, it would be interesting to determine if this is a conserved mechanism for all NT-related genes and possibly other molecules associated with more differentiated neuronal features, such as ion channels.

Interestingly, the timing of translation initiation of the analysed NT pathway genes is close to the beginning of synaptogenesis, which is described to start around 60 h APF in the CB [17] and around 55 h APF in the optic lobe [15,19,66]. These timings suggest that the beginning of translation could precede synaptogenesis, which might be required to ensure that neurons are ready to synchronously establish synapses.

Our results reveal a novel layer of posttranscriptional regulation involved in neuron maturation, opening the field to a whole set of new questions. As VGlut, ChAT, and Gad1 protein are only expressed several hours after their transcripts are present, why is their mRNA transcribed so much sooner? And how is their translation being inhibited? And how is it then initiated? We show that the regulation of NT gene translation timings occurs in an ecdysone-independent manner, as inhibiting ecdysone pathway using an EcRDN or by ablating ecdysone synthesis was not sufficient to induce premature translation of VGlut in young CB neurons during larval stages, nor to prevent VGlut translation in pupal stages. Ecdysone is known to coordinate the growth and developmental transitions of the animal; however, it does not seem to be involved in the coordination of NT gene translation with brain development. Another possibility is that NT gene translation is coordinated with the stimulus-independent neuronal activity that occurs just prior to synaptogenesis begins [66]. This would be an elegant mechanism to keep neurons primed for maturation, until the time comes when their synaptic partners are formed and ready to connect. At the cellular level, translation of NT-related genes could be inhibited by RNA binding proteins or translation inhibitors differentially expressed between Phases 1/2 and Phase 3 of neuron maturation. Supporting this hypothesis, our dataset discloses several RNA biding molecules and translation inhibitors—*Syncrip* (*Syp*), *musashi* (*msi*), *pumilio* (*pum*), *brain tumor* (*Brat*), or *polyA-binding protein interacting protein 2* (*Paip2*)—to be expressed in Phase 1 and Phase 2 neurons (**S6 Fig**). Interestingly, *pum* and *brat* are known to be involved in the maternal-to-zygotic transition, which is also dependent on posttranscriptional regulation and translation inhibition [87,88]. Although we did not explore this hypothesis, it would be interesting to test some of these candidates regarding their possible role in inhibiting the translation of NT genes. Interestingly, the important role of posttranscriptional regulation has already been described in mammalians, with aspects as RNA splicing, localization, and translation playing important roles in neural development. Moreover, many RNA-binding factors are associated with neurodevelopmental impairments in the cortex, further highlighting the importance of studying such mechanisms [89].

Overall, a mechanism of temporal regulation of translation may provide an efficient responsive mechanism to ensure that protein synthesis can quickly initiate for the final phase of neuron maturation and synapse formation. Ultimately, the identification of these 3 different phases of neuronal maturation represents an important foundation for further studies to understand the mechanism and timelines that regulate neuronal maturation.

In addition, this dataset of larva CB and VNC neural lineages represents a valuable resource for studying the regulatory genes and networks involved in NSC proliferation, lineage differentiation, and neural cellular diversity.

## Materials and methods

### Fly strains

For the scRNA-Seq experiments, temporal labeling of NBs and their lineages was achieved by crossing VT012709-Gal4 (VDRC; enhancer of Koi and CG15236; CB/VNC NB driver) with UAS-CD8::GFP; tub-Gal80ts males. Fly crosses were set up at 25°C, allowing approximately 12-h egg lays. First instar larvae (L1) were synchronized upon hatching and transferred to 18°C to inactivate CD8::GFP expression. Animals were shifted to 25°C, to activate CD8::GFP expression, 18 h before the dissection time point at 105 h ALH.

For the permanent labeling experiments, males from the G-TRACE stock line;; UAS-*FLP*, *Ubi-p63E*-STOP-*Stinger* (Bloomington #28282) were crossed with females of either the tubGal80ts; VT201094-Gal4 or the *elav*-Gal4; tubGal80ts driver (G-TRACE, control). For the EcRDN experiments, males from; EcRDN;UAS-*FLP*, *Ubi-p63E*-STOP-*Stinger* (EcRDN;G-TRACE) were crossed with females of either the tubGal80ts; VT201094-Gal4 or the *elav*-Gal4; tubGal80ts driver line. The progeny of these crosses was synchronized upon hatching (L1) and transferred to 18°C, as described above. These animals were then transferred to 25°C to activate CD8::GFP expression at 50 h or at 87 h ALH, and clones were allowed to develop for 18 h or 72 h at 25°C. All developmental times refer to the equivalent age of development at 25°C.

UAS-*dicer2*; *insc*-Gal4,UAS-CD8::GFP (gift from J. Knoblich) and Gad1-EGFP (Bloomington #59304) animals were either dissected as wL3 or synchronized as white pupae (0 h APF) and dissected at the appropriate age.

*VGlut*-*T2A*-Gal4 (Bloomington #84697) and *ChAT*-*T2A*-Gal4 (Bloomington #84618) were crossed with UAS-Stg::GFP, kept at 25°C, and wL3 progeny was dissected.

For the knockdown experiments, the *elav*-Gal4; tubGal80ts driver was crossed with UAS-Pomp RNAi (VDRC #100628 KK), UAS- Prosβ2 RNAi (VDRC #103575 KK), and UAS-Rpt5 RNAi (Bloomington #32422), or with W1118 (control; gift from A. Jacinto) and kept at 29°C; progeny was dissected as wL3. Efficiency of the RNAi lines in leading to protein accumulation caused by impaired proteasomal degradation activity had already been shown by Lőw and colleagues [75].

For the PGX experiment,;; *phm*-Gal4::tubGal80ts virgins were crossed with;; UAS-*Grim* males (gift from C. Mirth) or with UAS-*Stg*::GFP for the control and maintained at 18°C. The progeny was synchronized at white pupae (0 h APF) and shifted to 25°C to ablate the PG. Animals with the ablated PG (PGX) and the respective control were dissected at 48 h APF.

Please check the Key Resources Table (Table 1) for more complete information about the *Drosophila* lines used in this study.

**Table 1:** Resources table.

REAGENT or RESOURCE	SOURCE	IDENTIFIER
Antibodies		
Guinea pig anti-dpn (dilution 1:1,000)	gift from Juergen Knoblich [38]	RRID:AB_2568007
rat polyclonal anti-ase (dilution 1:200)	gift from Juergen Knoblich [90]	RRID:AB_2567568
mouse monoclonal anti-elav (dilution 1:100)	DSHB	RRID:AB_528217
mouse monoclonal anti-ChAT4B1 (dilution 1:500)	DSHB	RRID:AB_528122
Rabbit anti-DVGLUT-C-terminal (dilution 1:500)	gift from Hermann Aberle [91]	N/A
mouse monoclonal anti-Arm (dilution 1:50)	DSHB	RRID:AB_528089
chicken polyclonal anti-GFP (dilution 1:100)	Abcam	RRID:AB_300798
goat anti-guinea pig Alexa 647 (dilution 1:1,000)	Thermo Fisher Scientific	RRID:AB_2735091
goat anti-rabbit Alexa 647 (dilution 1:1,000)	Thermo Fisher Scientific	RRID:AB_2633282
goat anti-mouse Alexa 647 (dilution 1:1,000)	Invitrogen	RRID:AB_2535804
goat anti-rat Alexa 568 (dilution 1:1,000)	Thermo Fisher Scientific	RRID:AB_2534121
goat anti-mouse Alexa 568 (dilution 1:1,000)	Thermo Fisher Scientific	RRID:AB_144696
goat anti-chicken Alexa 488 (dilution 1:1,000)	Invitrogen	RRID:AB_142924
goat anti-mouse Alexa 405 (dilution 1:1,000)	Invitrogen	RRID:AB_221604
Chemicals, Peptides, and Recombinant Proteins		
aqua/polymount	Polysciences	N/A
formamide deionized	PanReac AppliChem	Cat. No. A2156
MG-132 [Z-Leu- Leu-Leu-CHO] (CAS 133407-82-6)	Santa Cruz Biotechnology	Cat. No. sc-201270
DMSO (dimethyl sulphoxide, CAS 67-68-5)	Sigma	Cat. No. D8418
Critical Commercial Assays		
DynaBeads MyOne Silane Beads	Thermo Fisher Scientific	Cat. No. 37002D
Chromium Single Cell 3′ Library and Gel Bead Kit v2, 4 rxns	10x Genomics	Cat. No. 120267
Chromium Single Cell A Chip Kit, 16 rxns	10x Genomics	Cat. No. 1000009
Stellaris RNA FISH Hybridization Buffer	BioSearch Tecnhologies	Cat. No. SMF-HB1-10
Deposited Data		
Single-cell RNA sequencing of *Drosophila melanogaster* Central Brain and Ventral Nerve Cord (raw and analyzed data)	this study	GEO: GSE179763
Experimental Models: Organisms/Strains		
Fly: *D*. *melanogaster*: w^1118^	gift from António Jacinto	N/A
Fly: *D*. *melanogaster*: VT012709-Gal4	gift from Juergen Knoblich	N/A
Fly: *D*. *melanogaster*: UAS-CD8::GFP; *tub*-Gal80ts	this study	N/A
Fly: *D*. *melanogaster*: *tub*-Gal80ts; VT012709-Gal4	this study	N/A
Fly: *D*. *melanogaster*: *elav*-Gal4; *tub*-Gal80ts;	this study	N/A
Fly: *D*. *melanogaster*: UAS-*FLP*, *Ubi-p63E*-STOP-*Stinger*	Bloomington Drosophila Stock Center	RRID:BDSC_28282
Fly: *D*. *melanogaster*: UAS-*dicer2*; *insc*-Gal4,UAS-CD8::GFP	gift from Juergen Knoblich	N/A
Fly: *D*. *melanogaster*: *Gad1*-EGFP	Bloomington Drosophila Stock Center	RRID:BDSC 59304
Fly: *D*. *melanogaster*:;UAS*-Stg*::GFP;	gift from Juergen Knoblich	N/A
Fly: *D*. *melanogaster*: *VGlut-T2A-*Gal4	Bloomington Drosophila Stock Center	RRID:BDSC 84697
Fly: *D*. *melanogaster*: *ChAT-T2A-*Gal4	Bloomington Drosophila Stock Center	RRID:BDSC 84618
Fly: *D*. *melanogaster*: UAS-*Pomp* RNAi	Vienna Drosophila Resource Center	VDRC ID#100628KK
Fly: *D*. *melanogaster*: UAS- *Prosβ2* RNAi	Vienna Drosophila Resource Center	VDRC ID#103575KK
Fly: *D*. *melanogaster*: UAS-*Rpt5* RNAi	Bloomington Drosophila Stock Center	RRID:BDSC_32422
Fly: *D*. *melanogaster*: EcRDN; UAS-*FLP*, *Ubi-p63E*-STOP-*Stinger*	This study	N/A
Fly: *D*. *melanogaster*:*;;phm-*Gal4::*tub*Gal80ts	gift from Christen Mirth	N/A
Fly: *D*. *melanogaster*:*;;*UAS*-Grim*	gift from Christen Mirth	N/A
Oligonucleotides		
Stellaris FISH Probes for VGlut, Quasar 570 Dye	BioSearch Technologies (sequence as in [92])	N/A
Stellaris FISH Probes for ChAT, Quasar 570 Dye	BioSearch Technologies (designed using Stellaris Probe Designer; sequence in S3 Table)	N/A
Stellaris FISH Probes for Gad, Quasar 570 Dye	BioSearch Technologies (sequence as in [92])	N/A
Software and Algorithms		
R Statistical Computing Software version 4.0.0	N/A	https://www.r-project.org/
Fiji	[93]	https://fiji.sc/; RRID:SCR_002285
Cell Ranger	10x Genomics	https://support.10xgenomics.com/single-cell-dna/software/release-notes/1-1#header; RRID:SCR_017344
Seurat version 3.1.5	[41]	https://satijalab.org/seurat/; RRID:SCR_007322
Velocyto v.0.17.17	[59]	http://velocyto.org/; RRID:SCR_018167
Monocle version 2.16.0	[63]	http://cole-trapnell-lab.github.io/monocle-release/
PANTHER	[94,95]	http://pantherdb.org/; RRID:SCR_004869
Circlize version 0.4.9	[96]	http://cran.r-project.org/web/packages/circlize/; RRID:SCR_002141
GraphPad Prism	GraphPad Software	RRID:SCR_002798
Imaris	Oxford Instruments	RRID:SCR_007370

### Brain dissociation and cell sorting

One hundred and twenty-four third instar larvae (105 h ALH) were collected and dissected in supplemented Schneider’s medium (10% fetal bovine serum (Sigma), 20 mM Glutamine (Sigma), 0.04 mg/mL L-Glutathione (Sigma), and 0.02 mg/mL Insulin (Sigma) Schneider’s medium (Sigma)). After dissection, brains were transferred to Chan & Gehring solution [97] 2% FBS and washed once. After this, they were enzymatically dissociated in Chan & Gehring solution 2% FBS with 1 mg/mL Papain (Sigma) and 1 mg/mL Collagenase I (Sigma) for 1 h at 30°C. Afterwards, brains were washed once with Chan & Gehring 2% FBS solution and once more with supplemented Schneider’s medium. After these washing steps, brains were resuspended in PBS (phosphate buffered saline) 0.1% BSA (Sigma) and mechanically disrupted using a pipette tip. The cell suspension was filtered through a 30-μL mesh into a 5-mL FACS tube (BD Falcon) and immediately sorted by fluorescence activated cell sorting (FACS) (FACS Aria II, BD). GFP-positive NBs and their lineage were collected in a drop of PBS 0.1% BSA. Since NBs represent a lower percentage of sorted cells when compared to neurons, they were sorted separately in order to assure an enrichment of less differentiated cells in the final pool. Cells were resuspended in 0.1% BSA at a final concentration of approximately 400 cell/μL and immediately processed according to the 10x Genomics protocol.

### 10x Genomics experimental procedure to generate CB/VNC dataset

Approximately 25k of the sorted cells (NB lineages) were used to construct single-cell libraries; libraries were obtained using Chromium Single Cell 3′ reagent Kits v2 (10x Genomics) standard protocol. Cells were equally divided into 2 samples (duplicates) and loaded in 2 wells of a Single Cell A Chip, aiming for an estimated target cell recovery of approximately 7k cells. Cells were then partitioned into nanoliter-scale Gel Bead-In-EMulsions (GEMs) and reverse-transcribed using an Eppendorf Mastercycler pro Thermal Cycler (Eppendorf), set for 53°C during 45 min, 85°C for 5 min and hold at 4°C. Post-reverse transcription incubation GEMs were then broken, and the cDNA was recovered and cleaned using Silane DynaBeads (Thermo Fisher Scientific). The next step consisted in amplifying the cDNA, by incubating the samples in a Thermal Cycler programmed for 98°C during 3 min, 10 cycles of 98°C for 15 s, 67°C for 20 s, and 72°C for 1 min, followed by 72°C for 1 min and hold at 4°C. The amplified cDNA was then cleaned using SPRIselect and quantified using a TapeStation (Agilent Technologies). The amplified cDNA was fragmented, end-repared, and A-tailed by incubating in a Thermal Cycler at 32°C for 5 min, 65°C for 30 min, and hold at 4°C; next, the cDNA went through a double-sided size selection using SPRIselect. Subsequently, the samples went through adaptor ligation, by incubating in a Thermal Cycler at 20°C for 15 min, after which there was a new SPRIselect cleanup step. Afterwards, samples were attributed independent indexes and amplified by PCR using a Thermal Cycler set for 98°C for 45 s, 14 cycles at 98° for 20 s, 54°C for 30 s, and 72°C for 20 s, followed by 72°C for 1 min and hold at 4°C. The generated library went through a new double-sided size selection by SPRIselect and run on a TapeStation for quality control and quantification.

Both samples were subjected to paired-end sequencing using the NovaSeq 6000 system (Genome Technology Center at NYU Langone Health).

Quantification and quality of cDNA and respective libraries generated for 10x Genomics Data were assessed with TapeStation following the standard protocol available at https://www.agilent.com/cs/library/usermanuals/public/ScreenTape_HSD5000_QG.pdf (Agilent Technologies).

### scRNA-Seq raw datasets (CB/VNC dataset)

Each sequenced sample was processed with Cell Ranger Version 3.0.1 for alignment, barcode assignment, and UMI counting. Samples were mapped to BDGP6 reference genome from Ensembl.

### scRNA-Seq dataset preprocessing (CB/VNC dataset)

The filtered gene matrices obtained after Cell Ranger processing were analyzed with R package Seurat 3.1.5. Only cells that had at least 200 unique feature counts were included in the analysis. Moreover, we only kept cells with a percentage of mitochondrial genes inferior to 20%. Higher percentages of mitochondrial genes are usually indicative of cell damage/rupture and, consequently, of altered overall transcriptional content [58]. These initial quality control steps resulted in a dataset with 12,671 cells and 10,250 genes.

Samples were normalized using the NormalizeData function, and the top 2,000 most variable features were then identified using the FindVariableFeatures function. Next, ScaleData was used to scale all genes; within this step, the percentage of mitochondrial genes was also regressed out, in order to avoid artefacts in subsequent analysis.

### scRNA-Seq dataset clustering (CB/VNC dataset)

We performed a principal component analysis (PCA), using the previously calculated top 2,000 most variable genes. Next, we used an elbow plot and a jackstraw approach to identify significant PCs. For the complete dataset analysis, we used the first 51 PCs, as not to include PCs that were not significant (*p* > 0.05). Within the FindClusters function, the resolution parameter was set to 1.55 as it resulted in a granularity that allowed the identification of smaller cell populations such as type II NBs and INPs. The combination of these parameters originated 39 clusters.

Clusters were annotated into major groups corresponding to the different cell types identified in our dataset, resulting in 10 major clusters. This annotation was performed based on well-described markers for each cell type (**Figs 1E and S2C**), as well as based on relative cell localization within the UMAP plot.

### Differential expression between clusters (CB/VNC dataset)

We used Seurat to identify the specific markers for each cluster (**S3B Fig**). For that, we used the receiver operating curve (ROC) test to find the differentially expressed genes between clusters. Within that analysis, we selected an AUC (area under the ROC curve) >0.5, to assure that the only hits were from genes with predictive values to classify that cluster. Moreover, to assure that none of the hits is a scarcely expressed gene, we only considered genes expressed in at least 25% of the cells of either one of the groups compared. We established that the average log fold change between the 2 populations being compared should be higher than 0.25; moreover, the dot plots for the top cluster markers of these analysis only showed genes with a pct.1 > 0.5 and pct.2 < 0.2.

GO term enrichment analysis was performed in PANTHER with the statistical overrepresentation test for biological process.

### Quantification of cells expressing a gene (mRNA) in CB/VNC dataset

To identify the number of neurons expressing genes necessary for neurotransmitter biosynthesis (VGlut, VAChT, Gad1, Vmat), we determined the number of cells with more than 0 counts for each specific gene or combination of genes. Percentages were calculated in relation to the total number of neurons in the dataset.

### RNA velocity dynamics (CB/VNC dataset)

RNA velocity analysis was performed with the python version of Velocyto v.0.17.17 package [59]. We used the subcommand “velocity run” to create a loom file for the cells that survived the filtering steps of Seurat pipeline using the *Drosophila melanogaster* genome annotation file (Drosophila_melanogaster.BDGP6.88.gtf) and the bam file with sorted reads that was estimated using the default parameters of the Cellranger software (10x Genomics). We masked repetitive regions using the genome expressed repetitive annotation file downloaded from UCSC genome browser. The loom file created separates molecule counts into “spliced,” “unspliced,” or “ambiguous.” To estimate RNA velocity parameters, we adapted the pipeline used in the analysis of the mouse hippocampus dataset from La Manno and colleagues [57]. We started by removing cells with extremely low unspliced detection requiring the sum of unspliced molecules per cell to be above the 0.4 threshold. We also selected genes that are expressed above a threshold of total number of molecules in any of the clusters requiring 40 minimum expressed (spliced) counts in at least 30 cells, after which we kept the top 3,000 highly expressed and variant genes on the basis of a coefficient of variation (CV) versus mean fit that uses a nonparametric fit (Support Vector Regression). We applied a final filter to the dataset by selecting genes on the basis of their detection levels and cluster-wise expression threshold. This filter kept genes with unspliced molecule counts above a detection threshold of 25 minimum expressed counts detected over 20 cells, and with average counts of unspliced and spliced expression bigger than 0.01 and 0.08, respectively, in at least one of the clusters. Finally, both spliced and unspliced counts were normalized for the cell size by dividing this value by the total number of molecules in each cell and multiplying the mean number of molecules across all cells. All filtering steps resulted in a dataset of 12,604 cells and 1,086 genes to be used in the RNA velocyto analysis. For the preparation of the gamma fit, we smooth the data using a kNN neighbors pooling approach (velocyto subcommand knn_imputation) and k = 500 with calculations performed in the reduced PCA space defined by the top 99 principal components. Velocity calculation and extrapolation to future states of the cells were performed under the assumption of constant velocity. Analysis pipeline can be obtained from the corresponding author.

### Single-cell trajectories (CB/VNC dataset)

Pseudotime analysis was performed using R package Monocle v2.16.0. The Seurat object, containing all filtering and clustering information, was imported to Monocle; for the analysis of the neuronal population, all clusters annotated as “Neurons” were subset, and within those, only cells with 0 counts for *wor* and *repo* were processed.

Differences in mRNA across cells were normalized, and “dispersion” values were calculated using the functions estimateSizeFactors and estimateDispersions, respectively.

To construct single-cell trajectories, we started by using the differentialGeneTest function to extract the genes distinguishing different clusters; these genes were then marked to be used for clustering in subsequent calls by using setOrderFilter. Afterwards, dimensions were reduced by using the Discriminative Dimension Reduction Tree (DDRTree) method and, finally, ordered using the orderCells function.

We used differentialGeneTest to obtain the differentially expressed genes as a function of pseudotime. These genes were then ordered by qvalue, and the top 100 hits were presented.

### Gene annotation lists

The list for ion channels was obtained from Gene List Annotation for *Drosophila* (GLAD) [98], obtained from //www.flyrnai.org/tools/glad/web/.

For the cadherin super family analysis, we used a gene list from FlyBase, obtained under the group “CADHERINS”–FBgg0000105.

For the immunoglobulin super family analysis, genes were selected according to FlyBase FBrf0167517 [99].

In all 3 cases, gene symbols were updated according to our dataset. We used these gene lists to show the correspondence between cluster and cluster top 5 marker genes. To do this, we created chord diagrams using the R package “circlize,” adapted according to Allen and colleagues [31].

### Neurotransmitter temporal expression in optic lobe

To address the expression of VGlut, ChAT, and Gad1 throughout time, we used a previously generated dataset [18] that was downloaded from the Gene Expression Omnibus (GSE156455). Data referring to the DGRP (Drosophila Genetic Reference Panel) dataset were imported to Seurat, and standard protocol was followed as described by Kurmangaliyev and colleagues [18]. This dataset comprises time points from 0 h APF to 96 h APF with a 12-h interval (duplicates for each time point). QC and integration pipelines were performed as described in [18]. Samples from 48 h and 72 h were used as references with the FindIntegrationAnchors function, and the dimensionality was set to 200 in the IntegrateData function. The first 200 PCs were selected for further analysis, as well as a resolution of 10. Neuronal cells were annotated based on datasets from the adult animal [33,100]. Two types of neurons are shown for each of the represented neurotransmitter families: L5 and Mi1 (cholinergic), L1 and Mi1 (glutamatergic), Dm10 and Mi4 (GABAergic). The average transcriptional profile was calculated for each cell type (cluster) for each time point and for each replicate. Values are shown in TP10k (TP, transcripts per million).

### Immunofluorescence

Larval brains were dissected and fixed in 4% paraformaldehyde for 20 to 40 min at room temperature; afterwards were washed 3 times with PBS with 0.1% Triton X-100 (PBT). Fixed brains were incubated for 20 min in PBS with 0.5% Triton X-100 and 1% Normal Goat Serum (blocking solution) and incubated with the primary antibodies overnight at 4°C. Next day, brains were washed 3 times, blocked 20 min, and incubated with the secondary antibodies for 2 h at room temperature. Afterwards, brains were washed 3 times, incubated 10 min in PBS, mounted in aqua/polymount (Polysciences), and imaged.

Immunofluorescent images were acquired on a LSM880 (Carl Zeiss GmbH); the tile function (15% overlap) was used when necessary. Fiji was used to adjust brightness and contrast.

### Immunofluorescence quantifications

Fluorescence intensity was measured in Fiji. For each brain, a specific area of interest was selected, and the mean fluorescence intensity was determined. Arm signal was measured in OL epithelia, while VGlut and ChAT signal was measured in a patch of medulla neurons where both glutamatergic neurons and cholinergic neurons are known to exist [65]. Mean fluorescence intensity was also measured in the background and subtracted to the value measured in the area of interest. Results are represented normalized to the mean of the control brains.

The volume where anti-VGlut expression was detected was measured in the EcR-DN and PGX experiments. Measurements were performed in Imaris (Oxford Instruments).

### smFISH

Larval or pupal brains were dissected and fixed in 4% paraformaldehyde for 40 min at room temperature; afterwards, brains were washed 4 times with PBS and incubated with 70% EtOH overnight. The following day, brains were washed for 5 min at 37°C with washing buffer (10% deionized formamide in 2× saline sodium citrate (SSC) solution). Afterwards, hybridization was performed by incubating the brains overnight at 37°C with hybridization buffer (10% deionized formamide in Stellaris Hybridization Buffer (BioSearch Technologies)) with 250 μm of the appropriate Quasar 570-labelled Stellaris DNA probes (BioSearch Technologies). smFISH probe library for ChAT was designed online using Stellaris Probe Designer, and the respective sequences are listed in **S3 Table**; smFISH probe libraires for VGlut and Gad1 were the same as used by [92].

The next day, brains were washed twice with washing buffer, first for 15 min at room temperature, and then for 1 h. Brains were washed a final time for approximately 20 min at room temperature with PBS and mounted in aqua/polymount (Polysciences) and imaged. smFISH images were acquired on a LSM880 (Carl Zeiss GmbH). Fiji was used to process the images: The GDSC plugin was used to apply a Laplacian of Gaussian and then further processed by applying a filter for Gaussian blur 3D.

### Quantification of smFISH spots

smFISH spot quantification was performed manually in Fiji as described next. Individual lineages were selected, and for each lineage, 2 projections were generated in order to facilitate spot visualization. Each projection comprised 10 slices, amounting to a total of 5 μm for each projection. The number of spots in each projection was then quantified manually and distributed to one of the following categories: (1) spots that are located in cells that are next to the NB (younger cells); and (2) spots located in all the other cells from that lineage (older cells/farther from NB).

### Proteosome inhibition using MG132

Proteosome inhibition was achieved as described in [73]. Briefly, MG132 (Santa Cruz Biotecnhology) diluted in di-dimethyl sulphoxide (DMSO, Sigma) was added to 5 mL of fly food at a concentration of 600 μM; for the control, DMSO alone was used. A total of 80 first instar W1118 larvae were used for each condition and developed at 25°C until wL3, when they were dissected.

### Statistical analysis

Statistical analysis was performed in GraphPad Prism (GraphPad Software). Statistical differences were determined using two-tailed paired Student *t* test or with 1-way ANOVA, using a Bonferroni’s multiple comparison test. Data are presented in scatter plots with bars (mean ± SEM); *p*-values < 0.05 were considered statistically significant.

## Supporting information

S1 Table Gene ontology analysis of genes differentially more expressed in less mature neurons (Phase 1, Cluster 2). Related to Fig 2.
(XLSX)

S2 Table Gene ontology analysis of genes differentially more expressed in more mature neurons (Phase 2, Cluster 1). Related to Fig 2.
(XLSX)

S3 Table Sequence of the probe libraries used for ChAT smFISH. Related to Fig 3. (XLSX)

S1 Data Quantification of the number of smFISH spots detected for VGlut, ChAT, and Gad1. Related to S4 Fig. (XLSX)

S2 Data Normalized expression of ChAT, VGlut, and Gad1 in cholinergic (L5 and Mi1), glutamatergic (L1 and Mi1), and GABAergic (Dm10 and Mi4) optic lobe neurons, ranging from 0 h APF to 96 h APF, with a 12-h interval. Related to S4 Fig. (XLSX)

S3 Data Mean fluorescence intensity measured in wL3 raised in food supplemented with MG132 diluted in DMSO, or in DMSO alone (control). Related to Fig 4. (XLSX)

S4 Data Quantification of VGlut and ChAT fluorescence intensity in wL3 optic lobe regions after independent knockdown of different subunits of the proteosome in optic lobe neurons. Related to S4 Fig. (XLSX)

S5 Data Quantification of the volume of VGlut expression per central brain lobe at approximately 48 h APF in control brains, EcRDN;G-TRACE brains, and PGX brains. Related to S5 Fig. (XLSX)

S1 Fig Type II neural lineages identified by scRNA-Seq in wandering larvae brain. Related to Fig 1. (**A**) Schematic representation of temporal strategy used to label neural lineages used in the scRNA-Seq experiment; 18-h clone induced at 87 h ALH and analysed at 105 h ALH (wandering third instar larvae); oldest neurons in clone are approximately 12.5 h. (**B**) Close-up of a type II neural lineage, outlined (posterior side of the central brain); Dpn (white), Ase (magenta), GFP (green), Elav (blue); scale bar, 10 μm. (**C**) Schematic representation of type II neural lineages; cells are colored by expression of markers as described; green outline indicates the cells in which VT201094-Gal4 drives GFP expression in a 18-h time window; frequency of cell division is indicated in hours. ALH, after larval hatching; dINP, developing INP; GMC; ganglion mother cells; imINP, immature INP; INP, intermediate neural progenitors; mINP, mature INP; NB, neuroblast; scRNA-Seq, single-cell RNA sequencing.
(TIF)

S2 Fig Transcriptomic profile of the larval lineages analysed. Related to Fig 1. (**A**) UMAP plot showing the uniform distribution of both samples used in the scRNA-Seq analysis; cells are colored by sample. (**B**) UMAP visualization of scRNA-Seq dataset composed of 12.7K cells of neural lineages from CB and central nerve cord (prior to cell type annotation); the 39 clusters are labeled by number; cells are colored by cluster. (**C**) Feature plots for neural identity markers of neural cells: *dpn*, *mira*, *klu*, *erm*, *pnt*, *Sp1*, *ase*, *wor*, *pros*, *elav*, *repo*. Cells are colored in the UMAP plot according to the expression of each marker. The scale represents gene expression levels (normalized counts). The data underlying this figure are contained within GEO database (accession number: GSE179763). CB, central brain; scRNA-Seq, single-cell RNA sequencing; UMAP, uniform manifold approximation and projection.
(TIF)

S3 Fig Neurons reveal diversity in identities. Related to Fig 2. (**A**) Velocity field projected in the UMAP plot; arrows indicate average velocity at a local level and predict the future cell state. The analysis of RNA velocities predicts the direction of lineage differentiation from the less differentiated NBs to the more differentiated neurons. (**B**) Top differentially expressed markers between each neuronal cluster and all remaining clusters; ion channels (blue) and genes integrating the immunoglobulin and cadherin super families (green) are highlighted. Outlines identify clusters 5 and 35, predicted to be the oldest/more mature neurons in the dataset. (**C**) Number of neurons expressing neurotransmitter-associated genes (*VGlut*, *VAChT Gad1*, *Vmat*) independently or simultaneously (counts >0); the respective percentages within the total neuronal population are indicated in bold. Each portrayed gene is associated with a different neurotransmitter: glutamatergic (*VGlut*), cholinergic (*VAChT*), GABAergic (*Gad1*), or monoaminergic (*Vmat*). The data underlying this figure are contained within GEO database (Accession number: GSE179763). *Gad1*, *glutamic acid decarboxylase 1*; NB, neuroblast; UMAP, uniform manifold approximation and projection; *VAChT*, *Vesicular acetylcholine transporter*; *VGlut*, *vesicular glutamate transporter*; *Vmat*, *Vesicular monoamine transporter*.
(TIF)

S4 Fig VGlut, ChAT, and Gad1 transcript and protein dynamics. Related to Figs 3 and 4. (A, B) 18-h clone induced at 87 h ALH and analysed at 105 h ALH (wL3). Oldest neurons in clone are approximately 12.5 h. (A) Schematic representation of temporal strategy used to induce clones in neural lineages. (B) smFISH against Gad1. Individual mRNA molecules are displayed as magenta dots; clone cells are labeled with nuclear GFP (green). Dashed line delimitates a neural lineage clone; pink arrowhead indicates the closest cell to the neuroblast where mRNA is visible; yellow asterisk identifies a neuroblast. Z-projection from 11 slices with 0.25-μm interval; scale bar, 5 μm. (C) Schematic representation of the smFISH spot classification regarding distance to the neuroblast. All spots detected within a specific lineage were classified according to their distance towards the NB (yellow) as: (1) spots in cells that are touching the NBs (green); and (2) all spots located in cells that are more than 1-cell distance away from the NB (magenta). (D) Quantification of the number of smFISH spots detected for VGlut (4 lineages; 2 brains), ChAT (12 lineages; 4 brains), and Gad1 (10 lineages; 4 brains). Data shown as mean ± SEM; statistical analysis was done using unpaired two-tailed *t* test; **P value < 0.01, ***P value < 0.001. The data underlying this figure are contained within S1 Data. (E) Temporal expression of Gad1-GFP under endogenous regulation. Expression was assessed at wL3 (dashed line delimitates the OL) and at 24 h APF (Z-projection from 4 slices with 1-μm interval; Gad1 expression is indicated with an arrow); scale bar, 50 μm. (F) Single-cell transcript expression pattern of *ChAT*, *VGlut*, and *Gad1* in OL neurons at indicated times in the X axis (times in hours APF). Two types of neurons are shown for each of the represented neurotransmitter families: L5 and Mi1 (cholinergic), L1 and Mi1 (glutamatergic), Dm10 and Mi4 (GABAergic). Expression patterns shown for these genes range from 0 h APF to 96 h APF, with a 12-h interval; levels of expression are represented in normalized values; the red dashed line indicates 24 h APF, the approximate onset of protein detection in the OL. The data underlying this figure were obtained using the dataset generated by [18] and is contained within S2 Data. (G) Quantification of VGlut and ChAT fluorescence intensity in wL3 OL regions when the indicated subunits of the proteosome are knocked down in optic lobe neurons. *elav*-Gal4 was used to drive the expression of UAS-Pomp RNAi, UAS-Prosβ2 RNAi, and UAS-Rpt5 RNAi. Mean fluorescence intensity of VGlut and ChAT was measured in medulla neurons (W1118, *n =* 8 brains; Pomp RNAi, *n* = 5 brains; Prosβ2 RNAi, *n* = 5; Rpt5 RNAi, *n* = 5). Data shown as mean ± SEM; statistical analysis was done using 1-way ANOVA, with a Bonferroni’s multiple comparison test; *p*-values < 0.05 were considered statistically significant; ns, not significant. The data underlying this figure are contained within S4 Data. ALH, after larval hatching; APF, after puparium formation; ChAT, choline acetyltransferase; Gad1, glutamic acid decarboxylase 1; NB, neuroblast; OL, optic lobe; smFISH, single-molecule fluorescence in situ hybridization; VGlut, vesicular glutamate transporter; wL3, wandering third instar larvae.
(TIF)

S5 Fig Ecdysone is not necessary to inhibit VGlut translation in larval stages. Related to Fig 4. (**A, B**) Immunofluorescence images for VGlut antibody staining (magenta); VT201094-Gal4;tubGal80ts was used to permanently label with GFP NB-derived lineages using G-TRACE; outlines indicate examples of GFP-positive lineage clones. Scale bar, 20 μm. (**A**) 18-h clones induced at 87 h ALH and analysed at 105 h ALH; oldest neurons in clones are approximately 12.5 h. (**A**) G-TRACE (Control) and (**A’**) EcRDN;G-TRACE. (**B**) 72-h clones induced at 87 h and analysed at 159 h ALH (approximately 48 h APF); oldest neurons in clone are approximately 66.5 h. (**B**) G-TRACE (Control) and (B’) EcRDN;G-TRACE. (**C**-**E**) *elav*-Gal4;tubGal80ts was used to drive EcR-DN expression in neurons from 87 h ALH for 18 h (analysis at 105 h ALH) or for 72 h (analysis at 48 h APF). VGlut antibody staining (white) (**C**, **D**) G-TRACE (Control); (**C’**, **D’**) EcR-DN;G-TRACE. Outlines indicate examples of areas where VGlut is expressed. Scale bar in **C**, **C’** = 20 μm. Scale bar in **D**, **D’** = 50 μm. (**E**) Quantification of the volume of VGlut expression per lobe at approximately 48 h APF in control brains (*n* = 8) and EcRDN;G-TRACE (*n* = 10). Data shown as mean ± SEM. (**F**, **G**) Evaluation of VGlut expression at 48 h APF after prothoracic gland ablation at 0 h APF. (**F**) Control brains; (**F’**) Brains with prothoracic gland ablated, PGX. Outlines indicate examples of areas where VGlut is expressed. Scale bar, 50 μm. (**G**) Quantification of the volume of VGlut expression per lobe at 48 h APF in control brains (*n* = 5) and PGX (*n* = 7); indicated values refer to mean ± SEM. Statistical analysis was done using unpaired two-tailed *t* test. The data underlying this figure are contained within S5 Data. ALH, after larval hatching; APF, after puparium formation; NB, neuroblast; VGlut, vesicular glutamate transporter.
(TIF)

S6 Fig Translation inhibitors and RNA binding proteins are expressed in the young neurons represented in the scRNA-Seq dataset. Related to Fig 4. Feature plots to visualize the expression of translation inhibitors and RNA binding proteins: *brat*, *pum*, *msi*, *Paip2*, and *Syp*. Cells are colored in a UMAP plot according to their expression of each marker. The scale represents gene expression levels (normalized counts). The data underlying this figure are contained within GEO database (accession number: GSE179763). *brat*, brain tumor; *msi*, musashi; *Paip2*, polyA-binding protein interacting protein 2; *pum*, pumilio; scRNA-Seq, single-cell RNA sequencing; *Syp*, Syncrip.
(TIF)

## Abbreviations

AD: activation domain
ALH: after larval hatching
APF: after puparium formation
Arm: Armadillo
Brat: brain tumor
CB: central brain
ChAT: choline acetyltransferase
DBD: DNA-binding domain
dINPs: developing INPs
EcR: ecdysone receptor
EcRDN: dominant negative form of the ecdysone receptor
Gad1: glutamic acid decarboxylase 1
GMC: ganglion mother cell
GO: gene ontology
INP: intermediate neural progenitor
msi: musashi
NB: neuroblast
NSC: neural stem cell
NT: neurotransmitter
Paip2: polyA-binding protein interacting protein 2
PG: prothoracic gland
pum: pumilio
QC: quality control
scRNA-Seq: single-cell RNA sequencing
smFISH: single-molecule fluorescence in situ hybridization
Syp: Syncrip
TF: transcription factor
UAS: upstream activation sequence
UMAP: uniform manifold approximation and projection
VGlut: vesicular glutamate transporter
VNC: ventral nerve cord
