# Supplementary figures and images for: Asynchronous transcription and translation of neurotransmitter-related genes characterize the initial stages of neuronal maturation in Drosophila
Source: PLoS Biol. 2023 May 19;21(5):e3002115. doi: 10.1371/journal.pbio.3002115 (PMC10234549; doi:10.1371/journal.pbio.3002115)

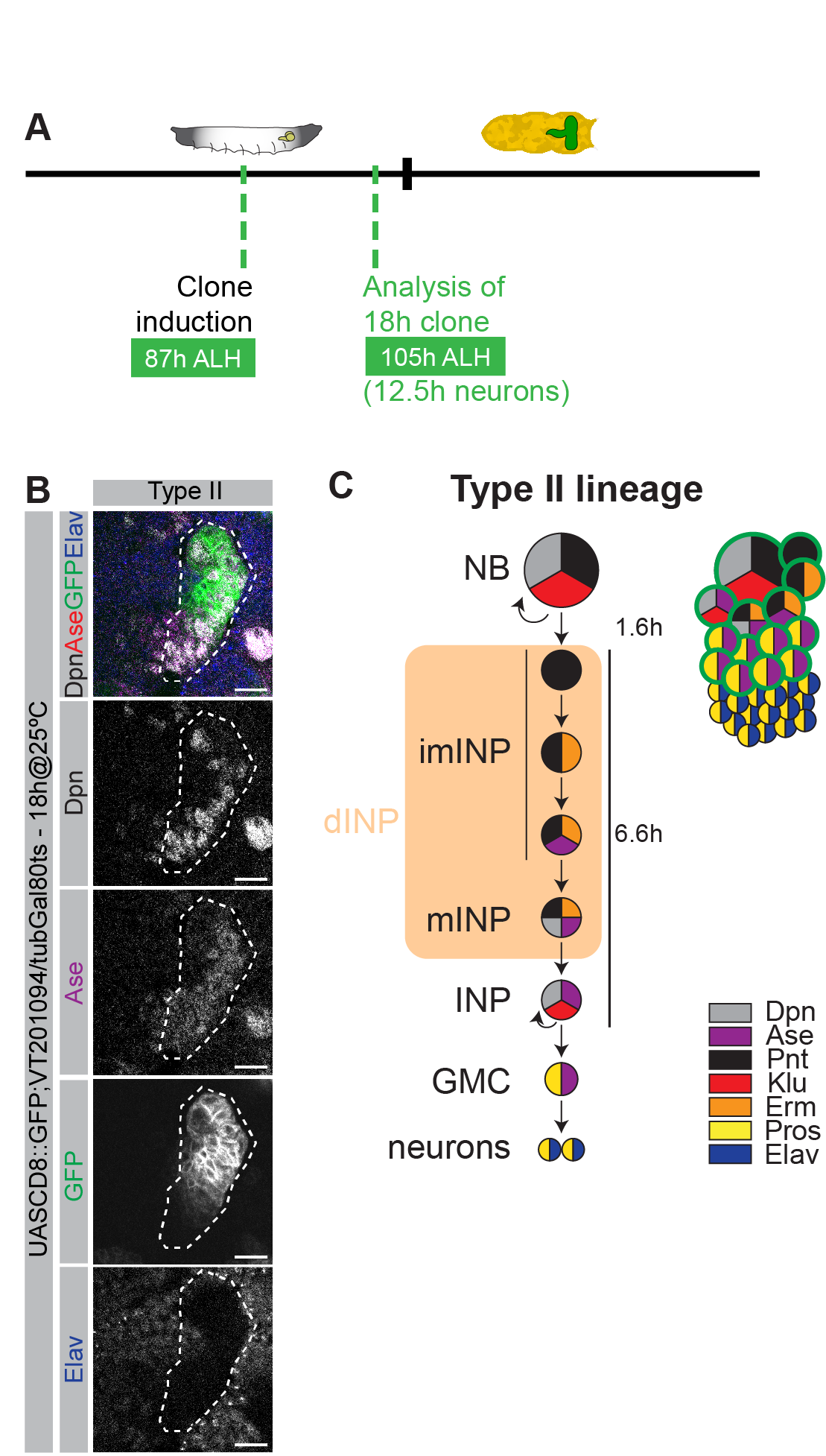

Supplement: S1 Fig — (A) Schematic representation of temporal strategy used to label neural lineages used in the scRNA-Seq experiment; 18-h clone induced at 87 h ALH and analysed at 105 h ALH (wandering third instar larvae); oldest neurons in clone are approximately 12.5 h. (B) Close-up of a type II neural lineage, outlined (posterior side of the central brain); Dpn (white), Ase (magenta), GFP (green), Elav (blue); scale bar, 10 μm. (C) Schematic representation of type II neural lineages; cells are colored by expression of markers as described; green outline indicates the cells in which VT201094-Gal4 drives GFP expression in a 18-h time window; frequency of cell division is indicated in hours. ALH, after larval hatching; dINP, developing INP; GMC; ganglion mother cells; imINP, immature INP; INP, intermediate neural progenitors; mINP, mature INP; NB, neuroblast; scRNA-Seq, single-cell RNA sequencing. (TIF) [file pbio.3002115.s009.tif]

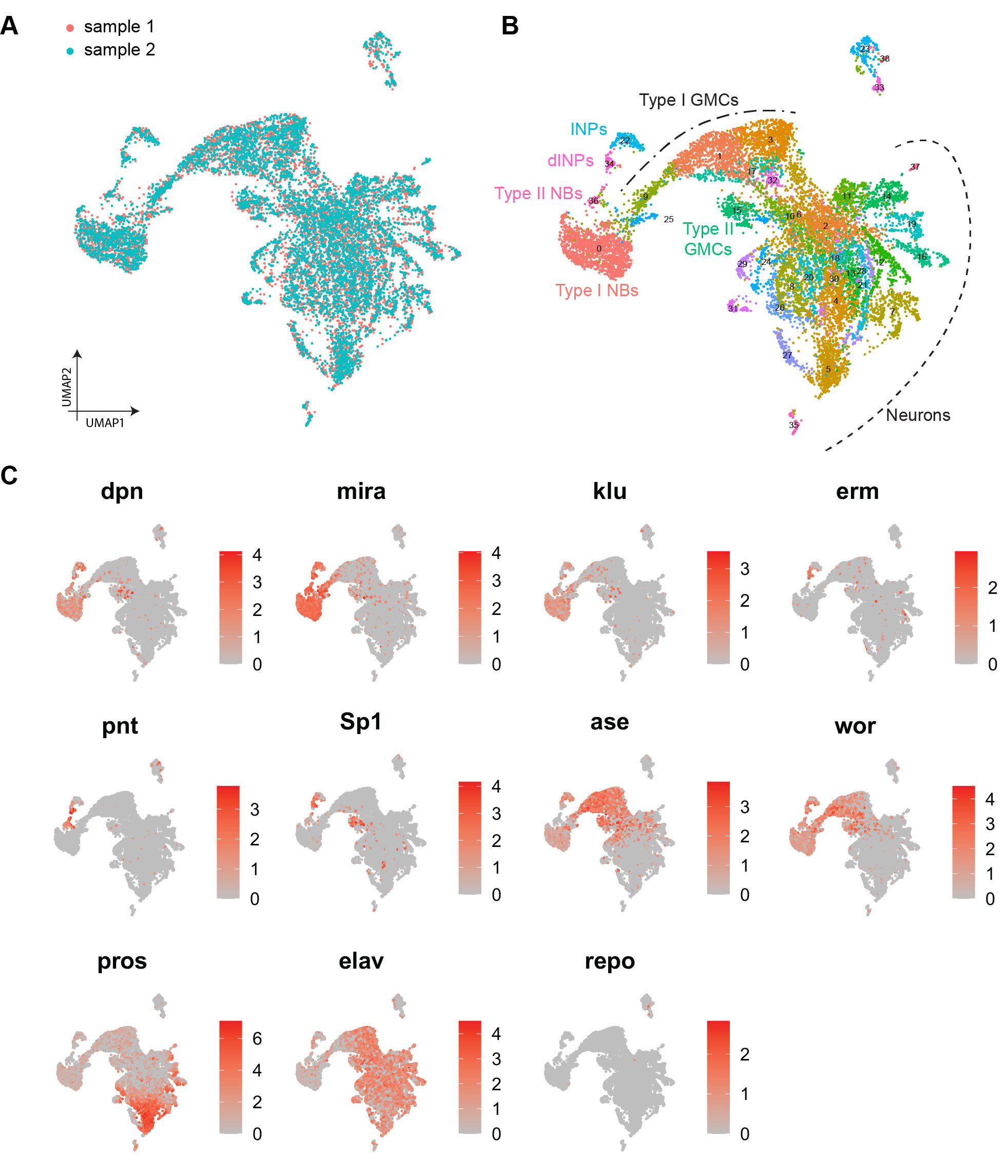

Supplement: S2 Fig — (A) UMAP plot showing the uniform distribution of both samples used in the scRNA-Seq analysis; cells are colored by sample. (B) UMAP visualization of scRNA-Seq dataset composed of 12.7K cells of neural lineages from CB and central nerve cord (prior to cell type annotation); the 39 clusters are labeled by number; cells are colored by cluster. (C) Feature plots for neural identity markers of neural cells: dpn, mira, klu, erm, pnt, Sp1, ase, wor, pros, elav, repo. Cells are colored in the UMAP plot according to the expression of each marker. The scale represents gene expression levels (normalized counts). The data underlying this figure are contained within GEO database (accession number: GSE179763). CB, central brain; scRNA-Seq, single-cell RNA sequencing; UMAP, uniform manifold approximation and projection. (TIF) [file pbio.3002115.s010.tif]

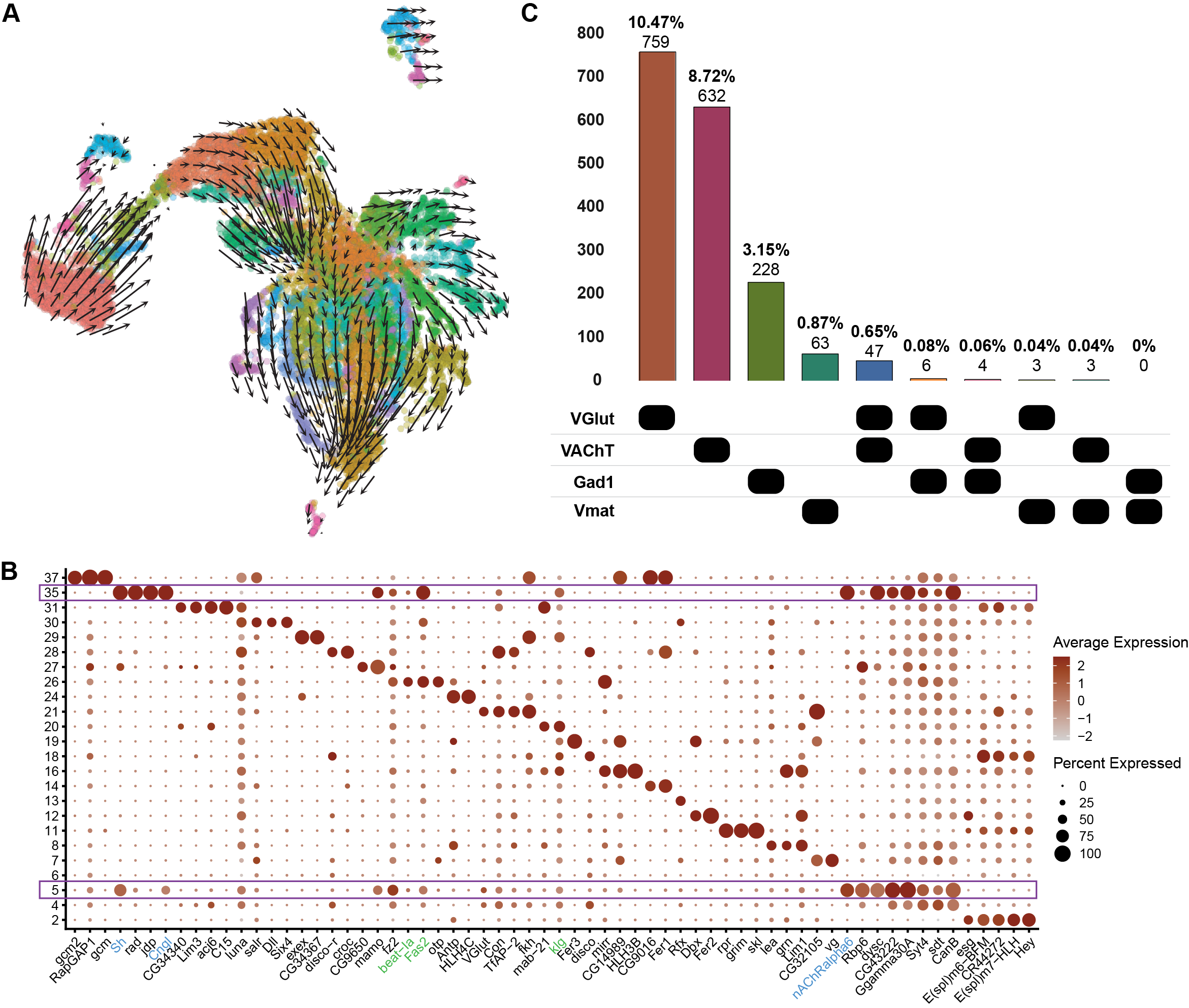

Supplement: S3 Fig — (A) Velocity field projected in the UMAP plot; arrows indicate average velocity at a local level and predict the future cell state. The analysis of RNA velocities predicts the direction of lineage differentiation from the less differentiated NBs to the more differentiated neurons. (B) Top differentially expressed markers between each neuronal cluster and all remaining clusters; ion channels (blue) and genes integrating the immunoglobulin and cadherin super families (green) are highlighted. Outlines identify clusters 5 and 35, predicted to be the oldest/more mature neurons in the dataset. (C) Number of neurons expressing neurotransmitter-associated genes (VGlut, VAChT Gad1, Vmat) independently or simultaneously (counts >0); the respective percentages within the total neuronal population are indicated in bold. Each portrayed gene is associated with a different neurotransmitter: glutamatergic (VGlut), cholinergic (VAChT), GABAergic (Gad1), or monoaminergic (Vmat). The data underlying this figure are contained within GEO database (Accession number: GSE179763). Gad1, glutamic acid decarboxylase 1; NB, neuroblast; UMAP, uniform manifold approximation and projection; VAChT, Vesicular acetylcholine transporter; VGlut, vesicular glutamate transporter; Vmat, Vesicular monoamine transporter. (TIF) [file pbio.3002115.s011.tif]

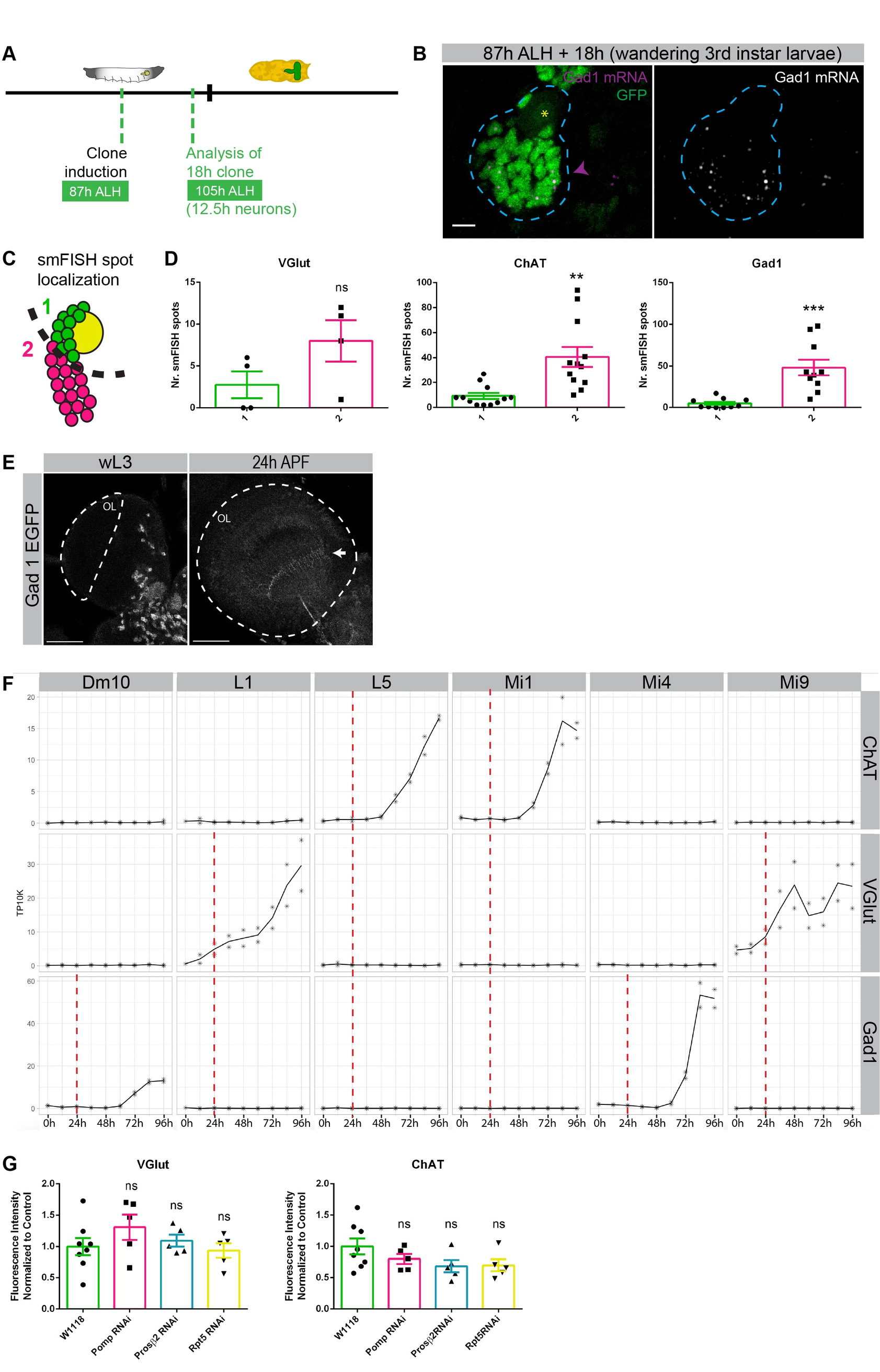

Supplement: S4 Fig — (A, B) 18-h clone induced at 87 h ALH and analysed at 105 h ALH (wL3). Oldest neurons in clone are approximately 12.5 h. (A) Schematic representation of temporal strategy used to induce clones in neural lineages. (B) smFISH against Gad1. Individual mRNA molecules are displayed as magenta dots; clone cells are labeled with nuclear GFP (green). Dashed line delimitates a neural lineage clone; pink arrowhead indicates the closest cell to the neuroblast where mRNA is visible; yellow asterisk identifies a neuroblast. Z-projection from 11 slices with 0.25-μm interval; scale bar, 5 μm. (C) Schematic representation of the smFISH spot classification regarding distance to the neuroblast. All spots detected within a specific lineage were classified according to their distance towards the NB (yellow) as: (1) spots in cells that are touching the NBs (green); and (2) all spots located in cells that are more than 1-cell distance away from the NB (magenta). (D) Quantification of the number of smFISH spots detected for VGlut (4 lineages; 2 brains), ChAT (12 lineages; 4 brains), and Gad1 (10 lineages; 4 brains). Data shown as mean ± SEM; statistical analysis was done using unpaired two-tailed t test; **P value < 0.01, ***P value < 0.001. The data underlying this figure are contained within S1 Data. (E) Temporal expression of Gad1-GFP under endogenous regulation. Expression was assessed at wL3 (dashed line delimitates the OL) and at 24 h APF (Z-projection from 4 slices with 1-μm interval; Gad1 expression is indicated with an arrow); scale bar, 50 μm. (F) Single-cell transcript expression pattern of ChAT, VGlut, and Gad1 in OL neurons at indicated times in the X axis (times in hours APF). Two types of neurons are shown for each of the represented neurotransmitter families: L5 and Mi1 (cholinergic), L1 and Mi1 (glutamatergic), Dm10 and Mi4 (GABAergic). Expression patterns shown for these genes range from 0 h APF to 96 h APF, with a 12-h interval; levels of expression are represented in n [file pbio.3002115.s012.tif]

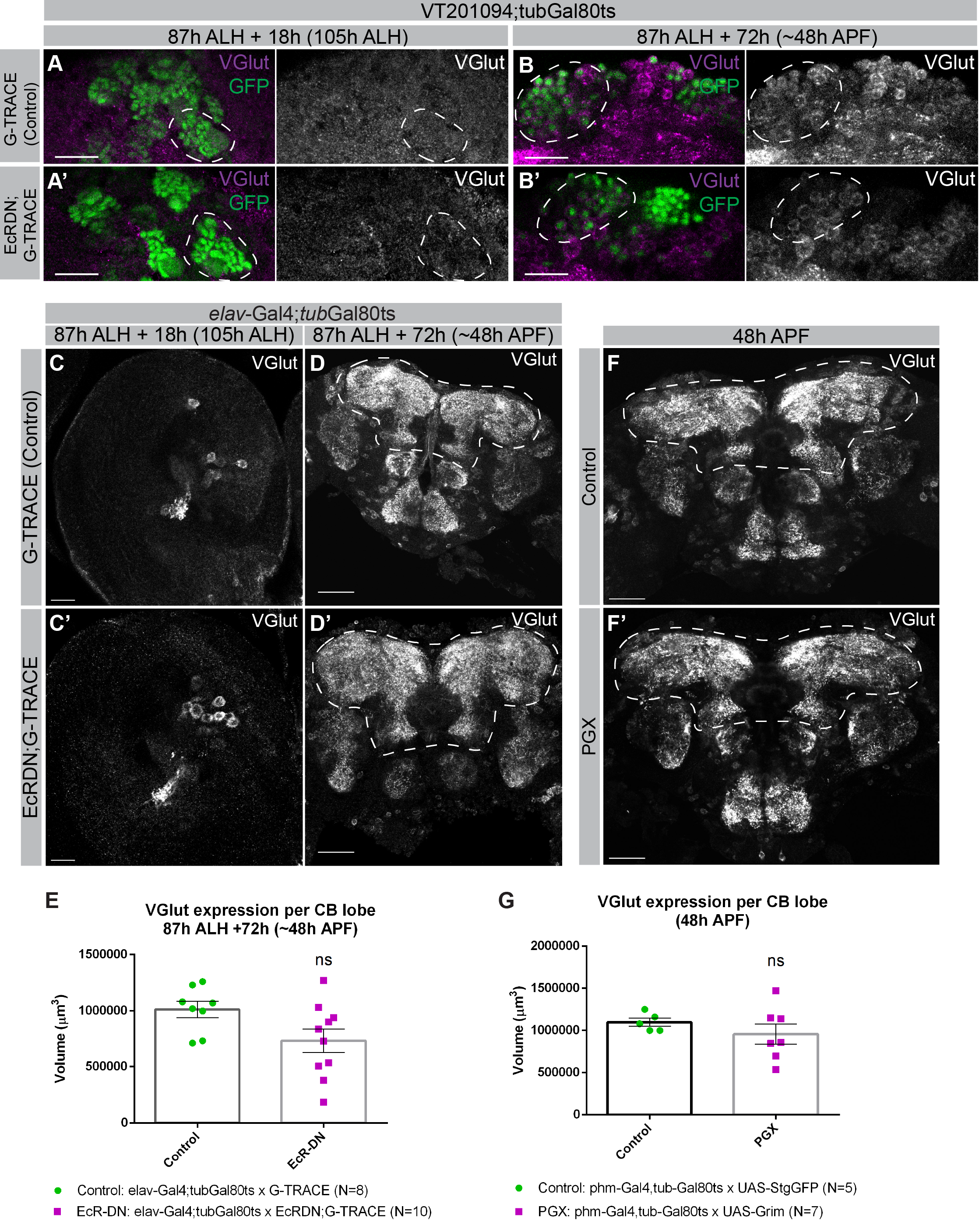

Supplement: S5 Fig — (A, B) Immunofluorescence images for VGlut antibody staining (magenta); VT201094-Gal4;tubGal80ts was used to permanently label with GFP NB-derived lineages using G-TRACE; outlines indicate examples of GFP-positive lineage clones. Scale bar, 20 μm. (A) 18-h clones induced at 87 h ALH and analysed at 105 h ALH; oldest neurons in clones are approximately 12.5 h. (A) G-TRACE (Control) and (A’) EcRDN;G-TRACE. (B) 72-h clones induced at 87 h and analysed at 159 h ALH (approximately 48 h APF); oldest neurons in clone are approximately 66.5 h. (B) G-TRACE (Control) and (B’) EcRDN;G-TRACE. (C-E) elav-Gal4;tubGal80ts was used to drive EcR-DN expression in neurons from 87 h ALH for 18 h (analysis at 105 h ALH) or for 72 h (analysis at 48 h APF). VGlut antibody staining (white) (C, D) G-TRACE (Control); (C’, D’) EcR-DN;G-TRACE. Outlines indicate examples of areas where VGlut is expressed. Scale bar in C, C’ = 20 μm. Scale bar in D, D’ = 50 μm. (E) Quantification of the volume of VGlut expression per lobe at approximately 48 h APF in control brains (n = 8) and EcRDN;G-TRACE (n = 10). Data shown as mean ± SEM. (F, G) Evaluation of VGlut expression at 48 h APF after prothoracic gland ablation at 0 h APF. (F) Control brains; (F’) Brains with prothoracic gland ablated, PGX. Outlines indicate examples of areas where VGlut is expressed. Scale bar, 50 μm. (G) Quantification of the volume of VGlut expression per lobe at 48 h APF in control brains (n = 5) and PGX (n = 7); indicated values refer to mean ± SEM. Statistical analysis was done using unpaired two-tailed t test. The data underlying this figure are contained within S5 Data. ALH, after larval hatching; APF, after puparium formation; NB, neuroblast; VGlut, vesicular glutamate transporter. (TIF) [file pbio.3002115.s013.tif]

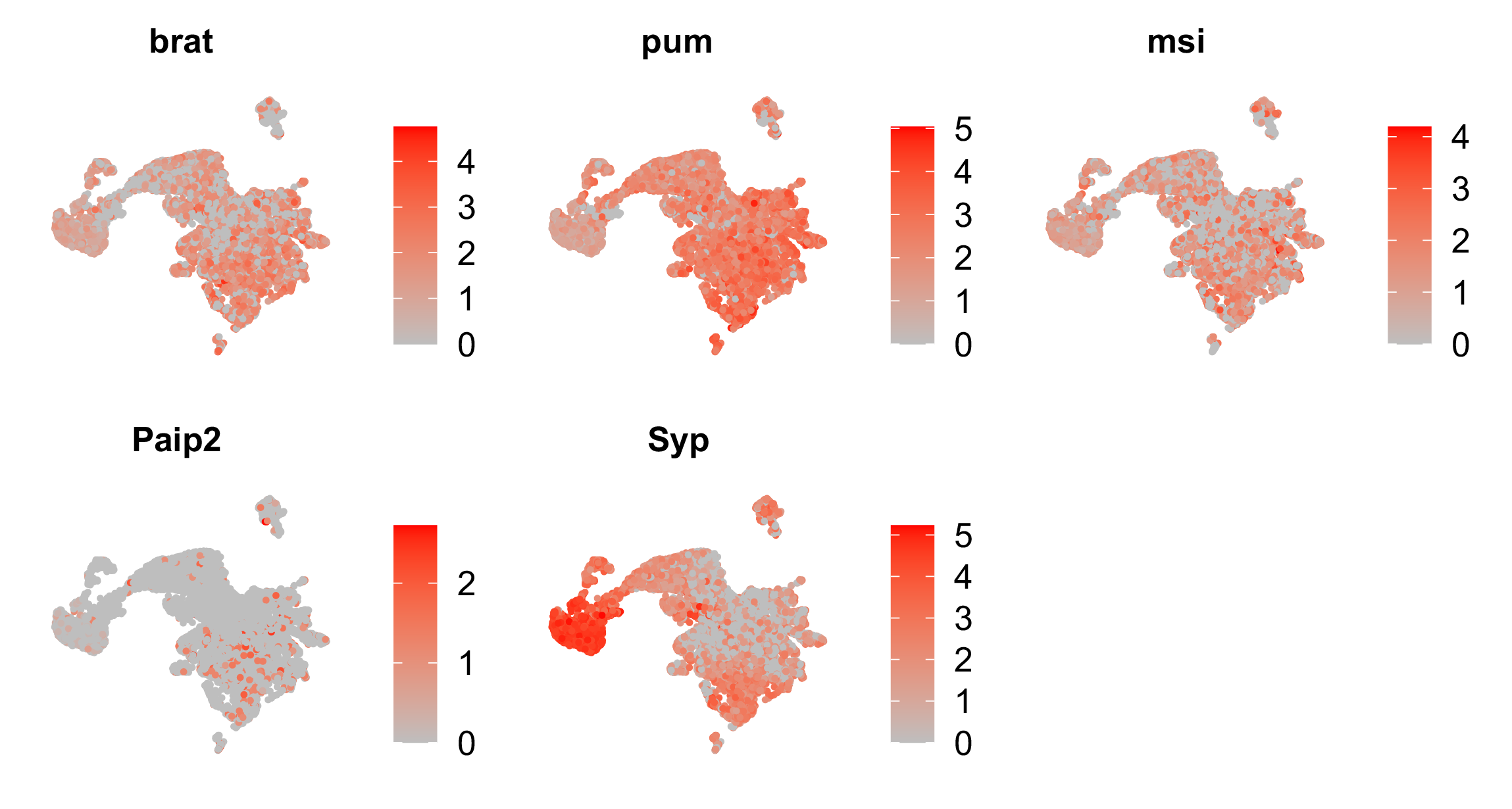

Supplement: S6 Fig — Feature plots to visualize the expression of translation inhibitors and RNA binding proteins: brat, pum, msi, Paip2, and Syp. Cells are colored in a UMAP plot according to their expression of each marker. The scale represents gene expression levels (normalized counts). The data underlying this figure are contained within GEO database (accession number: GSE179763). brat, brain tumor; msi, musashi; Paip2, polyA-binding protein interacting protein 2; pum, pumilio; scRNA-Seq, single-cell RNA sequencing; Syp, Syncrip. (TIF) [file pbio.3002115.s014.tif]
